# Temporal profiling of human lymphoid tissues reveals coordinated defense against viral challenge

**DOI:** 10.1038/s41590-024-02064-9

**Published:** 2025-01-31

**Authors:** Matthew L. Coates, Nathan Richoz, Zewen K. Tuong, Georgina S. Bowyer, Colin Y. C. Lee, John R. Ferdinand, Eleanor Gillman, Mark McClure, Lisa Dratva, Sarah A. Teichmann, David R. Jayne, Rafael Di Marco Barros, Benjamin J. Stewart, Menna R. Clatworthy

**Affiliations:** 1 Department of Medicine, Molecular Immunity Unit, University of Cambridge, Cambridge, UK; 2 Cambridge Institute for Therapeutic Immunology and Infectious Diseases, Cambridge, UK; 3 Cellular Genetics, Wellcome Sanger Institute, Hinxton, UK; 4 Cambridge Stem Cell Institute, Cambridge, UK

**Keywords:** Germinal centres, Mucosal immunology, Tonsils, Antimicrobial responses, Viral infection

## Abstract

Adaptive immunity is generated in lymphoid organs, but how these structures defend themselves during infection in humans is unknown. The nasal epithelium is a major site of viral entry, with adenoid nasal-associated lymphoid tissue (NALT) generating early adaptive responses. In the present study, using a nasopharyngeal biopsy technique, we investigated longitudinal immune responses in NALT after a viral challenge, using severe acute respiratory syndrome coronavirus 2 (SARS-CoV-2) infection as a natural experimental model. In acute infection, infiltrating monocytes formed a subepithelial and perifollicular shield, recruiting neutrophil extracellular trap-forming neutrophils, whereas tissue macrophages expressed pro-repair molecules during convalescence to promote the restoration of tissue integrity. Germinal center B cells expressed antiviral transcripts that inversely correlated with fate-defining transcription factors. Among T cells, tissue-resident memory CD8 T cells alone showed clonal expansion and maintained cytotoxic transcriptional programs into convalescence. Together, our study provides unique insights into how human nasal adaptive immune responses are generated and sustained in the face of viral challenge.

## Abstract (web summary)

Clatworthy and colleagues examine adult nasal lymphoid tissues in response to SARS-CoV-2 infection. Longitudinal profiling reveals changes in barrier tissue to block viral entry beyond the epithelial cell layer and how tissue repair occurred after viral infection.

## Main

Secondary lymphoid organs orchestrate the spatial and temporal arrangement of different immune cell subsets to enable appropriate cellular interactions and the generation of timely pathogen-specific responses. T and B lymphocytes are largely segregated into distinct areas within lymphoid tissues, except within germinal centers (GCs), highly organized structures with light and dark zones, where GC B cells acquire antigen from follicular dendritic cells (FDCs) and present it to PD^+^CD4^+^ T follicular helper (T_FH_) cells^1^. This interaction leads to iterative rounds of GC B cell proliferation, during which there is somatic hypermutation and the emergence of both memory B cells and antibody-secreting plasmablasts or cells that defend against current and future infection^1,2–7^. As lymphoid tissues are critical for the generation of adaptive immunity, they need robust defense mechanisms. These processes are well studied in animal models, for example, in mice, subcapsular sinus macrophages contribute to lymph node defense, preventing the spread of lymph-borne viruses and bacteria, the latter via interactions with innate lymphocytes^8,9^. In contrast, profiling of human lymphoid tissue responses during infection is limited, resulting partly from the challenges of sampling these structures in humans.

The nasopharynx contains the pharyngeal tonsils (also known as adenoids), which form part of Waldeyer’s ring of lymphoid tissue and are the major site of nasal-associated lymphoid tissue (NALT). Structurally, they have a surface crypt epithelium with antigen-transporting M cells and underlying B cell follicles and T cell-containing interfollicular areas, which together generate protective antibody-secreting and memory cells after a nasal challenge^10,11^. Adenoid tissue typically macroscopically involutes in adolescence and the extent to which NALTs contribute to local mucosal adaptive immune responses in adult humans is unclear. CD68^+^ macrophages have been identified in human adenoid NALT^12^, but macrophage heterogeneity and contributions to lymphoid tissue defense or repair post-infection in humans are completely unknown. The posterior nasal space can be readily visualized in humans using an endoscope, raising the possibility that endoscopic adenoid NALT biopsies might offer a feasible method to profile adaptive immune responses in secondary lymphoid organs after an upper respiratory tract immune challenge. To test this, we undertook a ‘natural experiment’, sampling adult participants after severe acute respiratory syndrome coronavirus 2 (SARS-CoV-2) infection—a virus that uses nasal epithelium as a point of entry and replication^13,14^—to understand how NALTs defend themselves in the face of local infection to ensure that the cells and infrastructure required to support an ongoing adaptive immune response are maintained.

Although SARS-CoV-2 has already been well studied in humans, much of this work has used peripheral blood or epithelial sampling in live participants, which fails to capture lymphoid follicular components, such as GC B cells^15–17^. Lymphoid tissue sampling in SARS-COV-2 infection has been conducted in children undergoing adenoidectomy, suggesting a robust GC response in pediatric NALT whereas, in contrast, autopsy samples from the spleen and lymph nodes in adults with fatal SARS-CoV-2 infection show minimal or absent GCs^18–20^.

In the present study, we applied single-cell RNA sequencing (scRNA-seq), multi-parameter flow cytometry and confocal imaging to adult NALT biopsies, to understand the cellular molecular adaptations that support, polarize and defend human secondary lymphoid tissue in adults in the face of a viral immune challenge.

## Results

### Postnasal space biopsy enables lymphoid tissue profiling

**Fig. 1:**
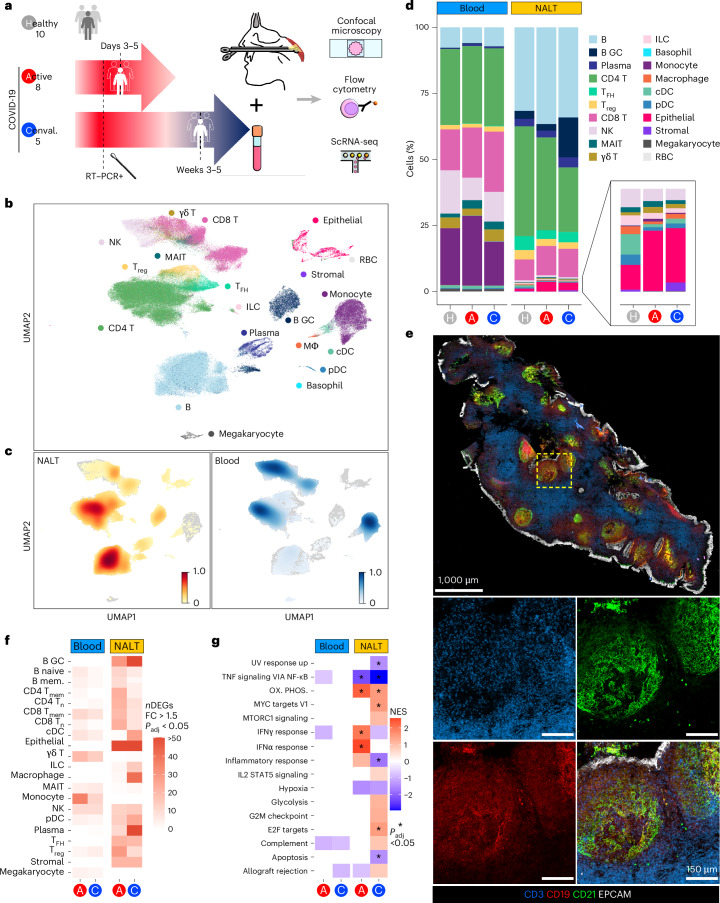
Experimental overview and cellular landscape of blood and NALT in SARS-CoV-2 infection. **a**, Schematic of experimental design with number of participants and timing of sampling in each group, sample types (paired NALT and peripheral blood) taken from participants and the techniques used to analyze processed samples. Conval., convalescent. **b**, Uniform Manifold Approximation and Projection (UMAP) of all cells in participants with COVID-19 and healthy controls, with major cell annotations. MΦ, macrophage; γδ T, gamma–delta T cell; RBC, red blood cell. **c**, UMAP of Scanpy embedding density (scanpy.tl.embedding_density), represented as a scaled Gaussian kernel density estimation, of all cells by sample type. NALT embedding density is shown in orange and blood embedding density in blue. **d**, Stacked bar charts showing proportional split (as percentage) of NALT and blood samples by cell type and disease type, with nested bar chart showing proportions, by disease type, of nasal NK cells, MAIT cells, γδ T cells, ILCs, monocytes, macrophages, cDCs, pDCs and epithelial and stromal cells. H, healthy control. **e**, Representative confocal images of a section of a postnasal space biopsy sample in a 19-year-old female from the convalescent COVID-19 group. The yellow dashed box indicates the region magnified in the four boxes in the lower part of the image panel (*n* = 1). **f**, Number of DEGs for disease versus healthy control samples in NALT and blood samples by fine cell-type cluster and disease type, where expression fold-change (FC) is >1.5 and Bonferroni’s adjusted *P* value (*P*_adj_) is <0.05. Two-sided Wilcoxon’s rank-sum test was used for computing DEGs. mem., memory. **g**, Hallmark GSEA of DEGs between disease participants and healthy controls for all (combined) cells by sample type and disease state. Ox. phos., oxidative phosphorylation. NES, normalized enrichment score: ^*^Benjamini–Hochberg *P*_adj_ < 0.05. NESs were calculated in the fgsea package with *P*-value estimation using an adaptive, multi-level, split Monte-Carlo scheme.

**Extended Data Fig. 1:**
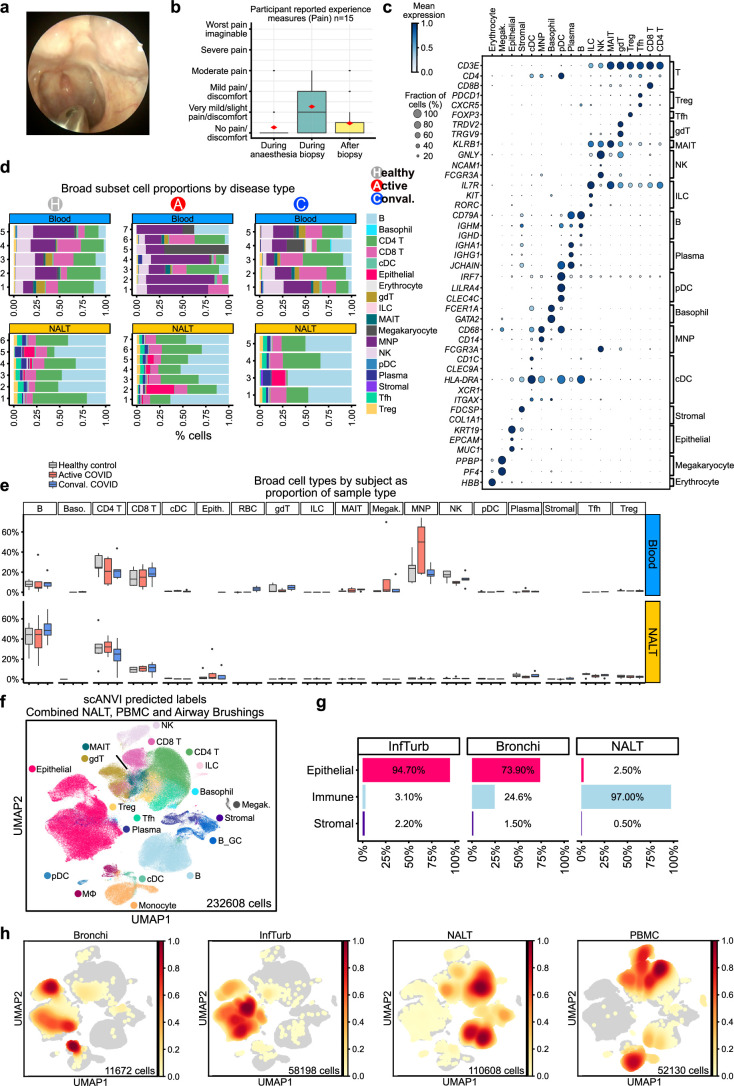
Experimental overview and cellular landscape of blood and NALT in SARS-CoV-2 infection. **a**, Endoscopic nasal view during an office-based postnasal space biopsy procedure in an awake human subject under topical local anaesthesia. Tilley-Henkel forceps are seen towards the bottom of the image. **b**, Boxplot of subject reported experience data during stages of local anaesthetic NALT biopsy procedure in 15 subjects. Median score represented by line, with red diamond showing mean score. **c**, Expression of canonical markers by assigned cell type label. Horizontal axis shows assigned broad cell labels, with genes grouped by canonical expression groups on vertical axis. Dot size shows percentage of gene expression by cells in group, with colour indicating normalised mean gene expression by cells in group. **d**, Stacked barplots showing cell type proportions by subject, condition and sample type. **e**, Serial boxplot showing cell type proportions by disease state, plotted by subject and sample. **f**, UMAP of scANVI integration of data and cell type labels from this paper with adult inferior turbinate and bronchial brushing data in SARS-CoV-2 subjects and controls previously published^2^. **g**, Proportional representation of airway sample data from e presented by sampling location and predicted cell type following scANVI integration. Cell type percentages are of total cells by sample location. **h**, Scanpy embedding density UMAP showing showing density of cells by sampling location on UMAP projection in **e**. Cell numbers by sample location are printed in the bottom right of the projection.

We developed a well-tolerated endoscopic technique for collecting NALT biopsies from live humans using topical local anesthesia (Extended Data Fig. 1a,b). 23 participants underwent sampling (aged 19–91 years), including 10 healthy controls, 8 patients with acute COVID-19 (sampled within 1 week of a first positive SARS-CoV-2 reverse transcriptase–polymerase chain reaction (RT–PCR) test) and 5 convalescent patients with COVID-19 (clinically asymptomatic participants sampled 3–5 weeks after the first positive SARS-CoV-2 RT–PCR; Fig. 1a and Supplementary Table 1). Postnasal space biopsies (with additional nasal brushing or curettage in some cases) were collected, alongside paired blood samples and processed for flow cytometry, scRNA-seq and confocal microscopy (Supplementary Table 1).

After quality control, we generated data on 162,738 cells and identified 20 major cell clusters across peripheral blood mononuclear cells (PBMCs) and nasal tissues, including epithelial cells, stromal cells and a variety of immune cell populations (Fig. 1b and Extended Data Fig. 1c–e). There was a significant overrepresentation of GC B cells and T_FH_ cells in nasal samples (Fig. 1c,d), reflecting the presence of GC-containing lymphoid structures. GC B cells were particularly abundant in convalescent NALT samples (Fig. 1d), consistent with an expansion of virus-specific B cells. NALT samples also differed from adult nasal cavity and bronchial brushings^17^, in which T_FH_ cells and GC B cells were scarce (Extended Data Fig. 1f–h). Confocal imaging of NALT biopsies confirmed the presence of B cell follicles containing CD21-expressing FDCs and spatially distinct T cell-rich areas, with overlying surface EPCAM^+^ epithelial cells (Fig. 1e). Notably, the number of differentially expressed genes (DEGs) in COVID-19 samples (both acute or active (A) and convalescent (C)), relative to controls, was greater in NALT than in blood (Fig. 1f). Similarly, gene-set enrichment analysis (GSEA) of these DEGs showed a greater magnitude, significance and number of enriched gene sets in NALT compared with peripheral blood, including interferon (IFN)α response pathway genes (Fig. 1g).

Overall, these data show that nasopharyngeal biopsies can be used to profile functional lymphoid tissue in adults, with capture of follicular cell types not found in blood or nasal cavity brushings. NALT sampling presents a feasible method to interrogate the cellular molecular processes occurring during the generation of adaptive immune responses to a nasal viral infection in humans.

### MNPs contribute to lymphoid tissue defense and repair

**Fig. 2:**
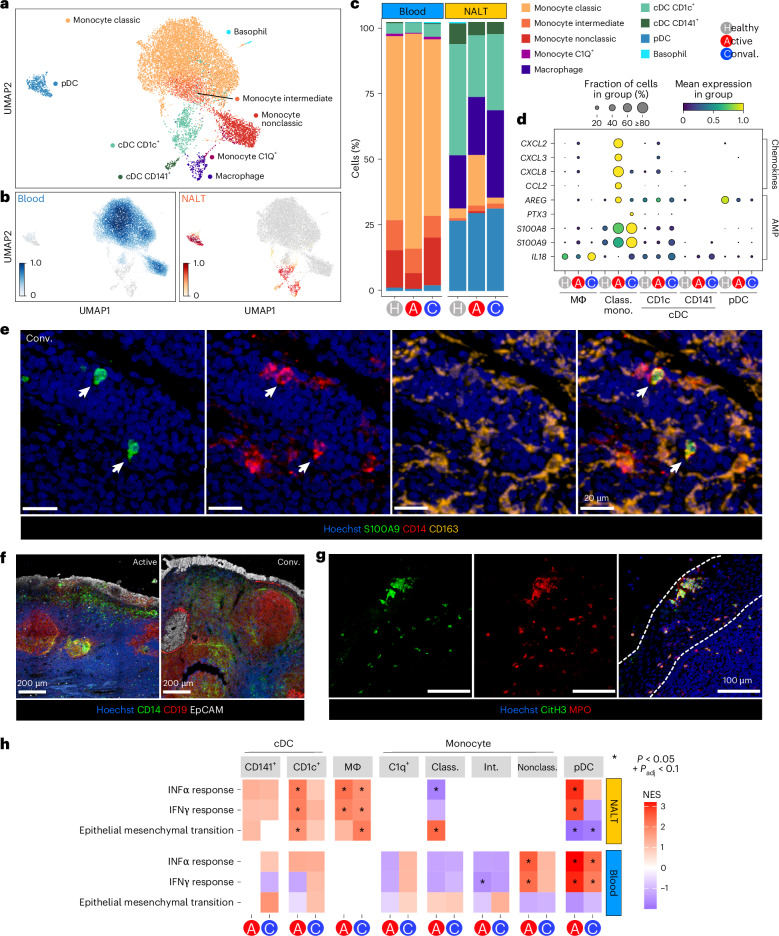
Recruited monocytes and inflammatory DCs form a defensive shield in the subepithelium. **a**, UMAP of MNPs reclustered in isolation, with assigned subset cell-type cluster labels shown. **b**, Scanpy embedding density (Gaussian kernel estimation) UMAP of reclustered MNP subset cell types showing scaled density of cells by sample type (blood or NALT) across the UMAP. NALT embedding density is shown in orange and blood embedding density in blue. **c**, Stacked bar chart showing proportional representation of MNP cell types in blood and nasal samples by disease status. **d**, Selected chemokine and antimicrobial peptide-scaled expression in NALT MNP subsets. Class. mono., classical monocyte. The size of the dot indicates the fraction of cells in the group expressing the gene and the color the scaled mean gene expression. **e**, Representative confocal imaging of section of NALT from a convalescent patient with COVID-19. The white arrowheads highlight the co-expression of S100A9 and CD14 (*n* = 1). **f**, Representative confocal imaging of sections of NALT from an active (left) and convalescent (right) patient (*n* = 2 per group). **g**, Representative confocal imaging of section of NALT from a patient with active COVID-19. The white dotted lines show the edges of the epithelium (*n* = 1). **h**, Heatmap of GSEA Hallmark gene-set enrichment for DEGs in MNP subsets by disease group against healthy control samples. Class., classical; Int., intermediate; Nonclass., nonclassical. The color of the tile shows the NES. Red shows increased gene-set enrichment and blue decreased gene-set enrichment relative to healthy controls, and white or no color shows no enrichment of the gene set. ^*^Statistical significance *P* < 0.05 and Benjamini–Hochberg’s *P*_adj_ < 0.1. NESs were calculated in the fgsea package with *P*-value estimation using an adaptive, multi-level, split Monte-Carlo schemeαγ.

**Extended Data Fig. 2:**
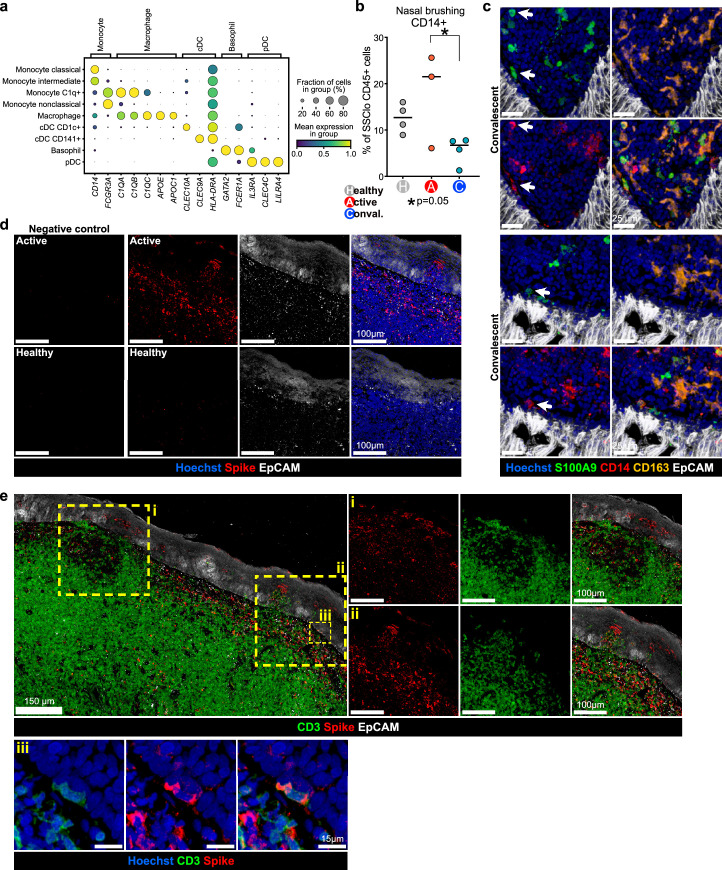
MNPs contribute to lymphoid tissue defence and repair. **a**, Expression of canonical markers by assigned MNP cell label for MNP cell subset. Vertical axis shows assigned broad cell labels, with genes grouped by canonical expression groups on horizontal axis. Size of point indicates fraction of cells in each group expressing corresponding gene, colour of point indicates scaled mean expression of corresponding gene in each named cell type group. **b**, Flow cytometry on inferior turbinate nasal brushing showing % of CD14+ cells in SSClo CD45+ live single cells. Each point represents a subject. Horizontal lines indicate median value. * p = 0.05 (Tukey HSD test). **c**, Confocal imaging of section of NALT from a convalescent COVID-19 subject. White arrowheads highlight co-expression of S100A9 and CD14. Related to Fig. 2e. n = 1. **d**, Representative confocal imaging of section of NALT from a healthy control and an active COVID-19 subject. n = 1 each. **e**, Confocal imaging of section of NALT from an active COVID-19 subject with indicated regions shown at higher magnification. n = 1.

**Extended Data Fig. 3:**
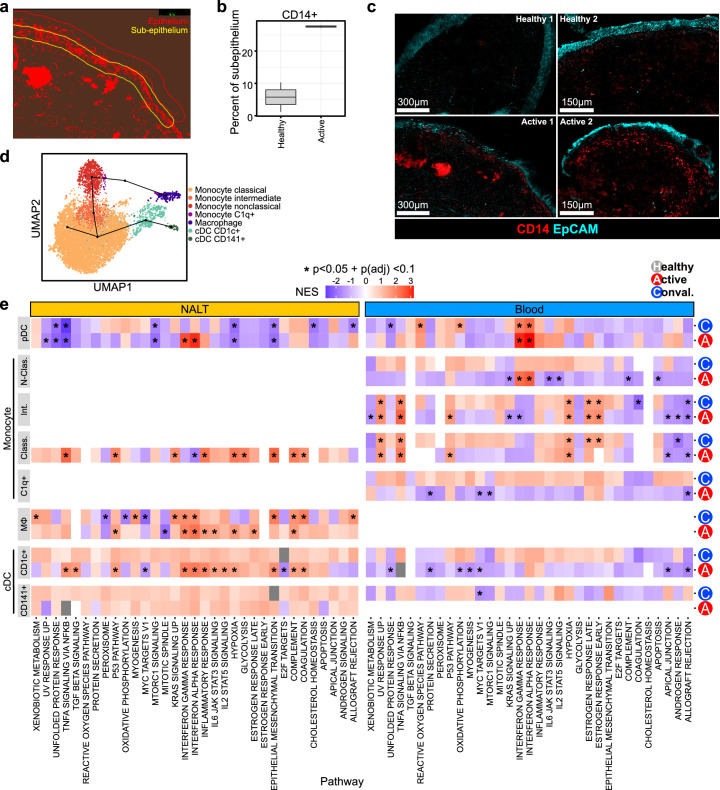
Monocytes localize at the subepithelium during acute SARS-COV-2 infection. **a**, Example of subepithelial marking strategy used in **b-c**. A 25 pt intensity thresholder has been applied to CD14 staining. Epithelium marked in dotted red line using EpCAM staining with fixed subepithelial area width marked in yellow dotted line. **b**, Barplot showing comparison of CD14+ staining in samples from **c** as a percentage of subepithelial region. **c**, Confocal imaging of sections of NALT from two separate healthy controls and active COVID-19 subjects. **d**, Slingshot pseudotime trajectories for MNP subset cell types, plotted in UMAP space. Black lines indicate Slingshot lineage trajectories, starting from Classical monocyte cluster. **e**, Heatmap showing all significantly enriched Hallmark pathways in MNP cell types following Gene set enrichment analysis of differentially expressed genes between COVID-19 disease groups and healthy control subjects. Only pathways containing at least one significant enrichment (p < 0.05 and Benjamini-Hochberg adjusted p value < 0.1) in MNP cell types are shown. Colour indicates normalised enrichment score (NES), with red indicating greater pathway enrichment in disease group compared with healthy controls, and blue indicating increased pathway enrichment in healthy controls than disease group.

Postnasal space sampling 3–5 d after diagnostic testing for SARS-CoV-2 provided a unique opportunity to understand how early innate immune responses act to defend NALT from local viral attack. Among mononuclear phagocytes (MNPs), we identified 11 cell types: conventional dendritic cells (cDCs) (*CD141*^+^ cDC1 and *CD1C*^+^ cDC2), plasmacytoid dendritic cells (pDCs), six monocyte clusters (classical, intermediate, nonclassical, *SIGLEC1*^+^, *CD163*^hi^ and *C1Q*^+^), basophils and macrophages (Fig. 2a and Extended Data Fig. 2a). The proportion of classical monocytes in both nasal tissue and peripheral blood increased in acute COVID-19 compared with healthy controls and convalescent COVID-19 (Fig. 2b,c), consistent with tissue infiltration (confirmed by flow cytometry; Extended Data Fig. 2b). These monocytes showed increased expression of several self- and neutrophil-recruiting chemokines (*CCL2* and *CXCL2*, *CXCL3* and *CXCL8*, respectively*)* (Fig. 2d). *S100A8/9* transcripts were also increased in classical monocytes in active and convalescent COVID-19 samples (Fig. 2d,e and Extended Data Fig. 2c). *S100A8*/9 dimerizes to form calprotectin, which sequesters metal ions, limiting their availability to bacteria, including upper respiratory tract-dwelling organisms such as staphylococci^21^. SARS-CoV-2 is known to directly infect respiratory tract epithelium and, indeed, we found SARS-CoV-2 spike protein in epithelial cells overlying NALT in acute COVID-19 (Extended Data Fig. 2d) and in some subepithelial T cells (Extended Data Fig. 2e). CD14^+^ monocytes predominantly localized to the subepithelium in acute COVID-19 (Fig. 2f and Extended Data Fig. 3a–c) and surrounded some B cell follicles in convalescent disease (Fig. 2f), forming a shield around them. In addition, neutrophils were also present in the epithelial and subepithelial areas, some with histone citrullination, required for formation of neutrophil extracellular trap (NET) structures that bind and kill invading bacteria^22^ (Fig. 2g). Therefore, calprotectin-producing monocytes and NET-losing neutrophils may work together to defend the underlying lymphoid tissues after epithelial cell damage by invading virus.

Macrophages were found in NALT but not in blood and were increased in convalescent COVID-19 samples. The number of DEGs in NALT-resident macrophages was also greater in convalescent COVID-19 compared with acute infection (Fig. 1f), including interleukin (IL)-18 (Fig. 2d), reminiscent of studies in murine lymph nodes showing macrophage production of IL-18 in the context of bacterial challenge^9^. It is interesting that a population of circulating *C1Q*^+^ monocytes was also expanded in convalescent COVID-19 (Fig. 2c and Extended Data Fig. 2a), with trajectory analysis suggesting that these represent tissue macrophage precursors (Extended Data Fig. 3d).

GSEA^23^ showed a greater magnitude of enrichment in nasal cell types compared with their circulating counterparts (Fig. 2h and Extended Data Fig. 3e). Considering the majority myeloid populations in NALT, ‘IFNα response’ and ‘INFγ response’ genes were significantly enriched in macrophages, *CD1C*^+^ dendritic cells (DCs) and pDCs in acute COVID-19 compared with controls (Fig. 2h and Extended Data Fig. 3e). In convalescent COVID-19, this IFN response gene enrichment became less prominent in cDCs and pDCs but was sustained in macrophages (Fig. 2h). Furthermore, ‘epithelial-to-mesenchymal transition’ (EMT) gene-set enrichment increased in macrophages in convalescent disease and, indeed, was exclusively enriched in macrophages at this time point (Fig. 2h and Extended Data Fig. 3e), consistent with a role in the restoration of tissue homeostasis.

**Fig. 3:**
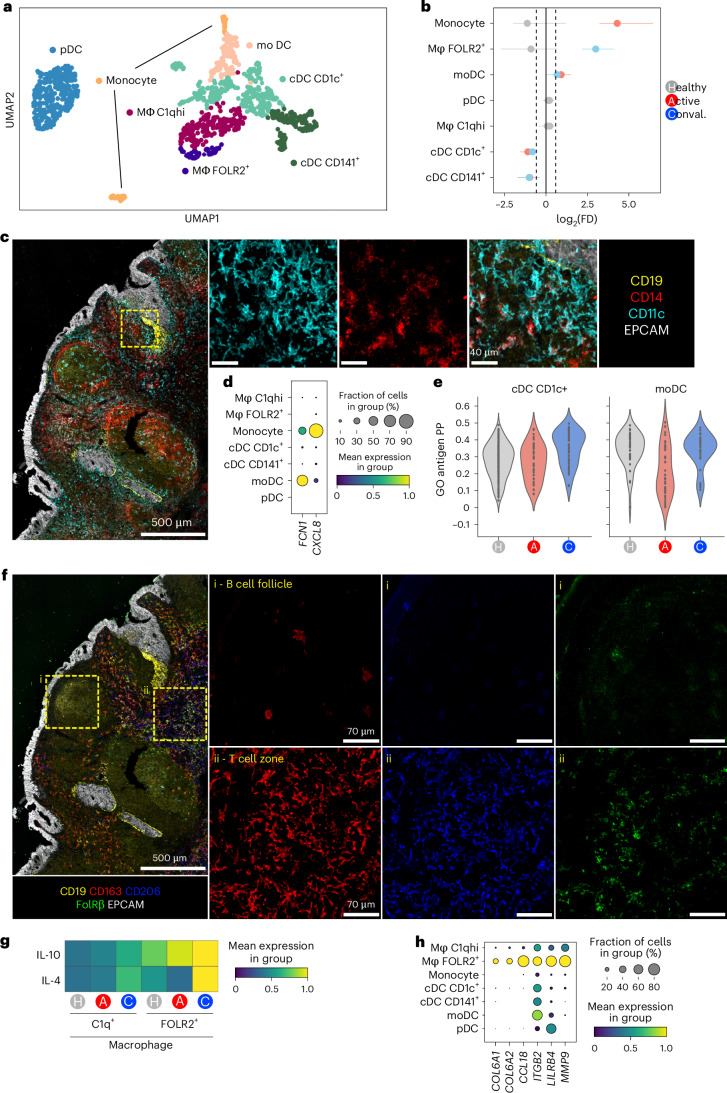
Tissue-resident macrophages take on pro-repair phenotype in convalescence. **a**, UMAP of NALT MNPs in isolation for participants with COVID-19 and healthy controls reclustered in isolation, with assigned subset cell-type cluster labels shown. **b**, Proportional difference in abundance of NALT MNP cell types in active COVID-19 (red) and convalescent COVID-19 (blue), relative to healthy controls. Nonsignificant values (*P* > 0.05) relative to healthy controls, following permutation testing, are shown in gray. The bars indicate the bootstrapped 95% CI. FD, fold distribution. **c**, Representative confocal imaging of a section of NALT from a convalescent COVID-19 participant. The yellow dashed box indicates a region magnified in the three boxes on the right-hand side (*n* = 1). **d**, Dotplot showing expression of *FCN1* and *CXCL8* in NALT MNP subsets. The size of the point indicates the fraction of cells in each group expressing the corresponding gene and the color the scaled mean expression of the corresponding gene in each named cell-type group. **e**, Violin plot showing expression of antigen processing and presentation gene ontology (GO) term genes (GO antigen PP) in conventional CD1c^+^ cDC compared with moDC cell subsets. Each point represents a cell. **f**, Representative confocal imaging of a section of NALT from a convalescent participant with COVID-19. The yellow dashed boxes indicate the regions magnified in the boxes on the right-hand side (*n* = 1). **g**, Heatmap indicating expression of genes associated with IL-4 or IL-10 stimulation in macrophage subsets, calculated as scaled gene-set expression scores^60^ using reference IL-10- or IL-4-stimulated macrophage gene sets^28^. **h**, Dotplot showing NALT MNP expression of *COL6A1*, *COL6A2*, *CCL18*, *ITGB2*, *LILRB4* and *MMP9*. The size of the point indicates the fraction of cells in each group expressing the corresponding gene and the color the scaled mean expression of the corresponding gene in each named cell-type group.

**Extended Data Fig. 4:**
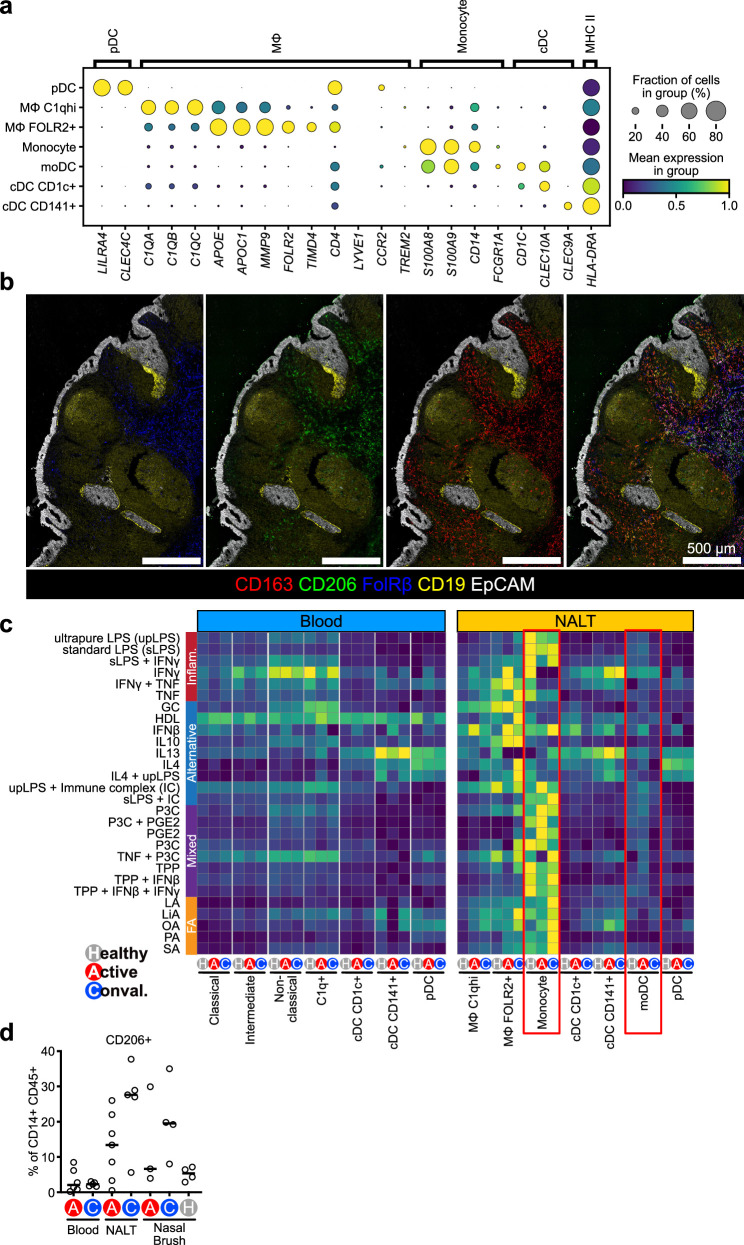
Tissue resident macrophages take on pro-repair phenotype in convalescence. **a**, Expression of canonical markers by assigned nasal MNP subset cell label. Horizontal axis shows assigned broad cell labels, with genes grouped by canonical expression groups on vertical axis. Size of point indicates fraction of cells in each group expressing corresponding gene, colour of point indicates scaled mean expression of corresponding gene in each named cell type group. pDC, plasmacytoid Dendritic Cell; Mφ, macrophage, moDC, monocyte-derived DC; cDC, conventional DC. **b**, Representative confocal imaging of section of NALT from a convalescent COVID-19 subject. Related to Fig. 3f. n = 1. **c**, Heatmap showing expression of genes associated with macrophage polarisation in all MNP subset cell types, split by disease type and sample type, calculated as scaled geneset expression scores (AddModuleScore^3^) using reference experimentally derived macrophage stimulation gene-sets^4^. Stimulation agent used for each gene-set shown on y-axis label, in correspondence to the original publication. Inflam., Inflammatory; FA, Fatty Acids. **d**, Column scatter graph showing percentage of CD206+ cells in live CD45 + CD14+ cells as determined by flow cytometry in blood, NALT and inferior turbinate nasal brushing samples. *, p(adj)<0.05 (Tukey HSD).

To further explore myeloid cell heterogeneity in NALT, we reclustered nasal MNPs in isolation from those in blood (Fig. 3a). This enabled the further identification of so-called ‘inflammatory’ or ‘monocyte-derived’ DCs (moDCs, based on the expression of *CD14*, *S100A8/9* and *FCGR1A*^24,25^ and two distinct clusters of macrophages based on *C1Q* and *FOLR2* expression; Fig. 3a and Extended Data Fig. 4a). The *FOLR2*^+^ macrophages expressed additional markers associated with tissue-resident macrophages, including *TIMD4* (ref. ^26^) (Extended Data Fig. 4a). Monocytes and moDCs were increased in acute COVID-19 (Fig. 3b). CD14^+^ moDCs localized to the subepithelial region (Fig. 3c) and expressed ficolin (*FCN1*) (Fig. 3d), a membrane c-type lectin that mediates CXCL8 upregulation on pathogen encounter^27^. A small proportion of moDCs also expressed *CXCL8* (Fig. 3d) and showed reduced enrichment of antigen presentation genes in acute COVID-19, in contrast to NALT cDCs (Fig. 3e). Altogether, this is consistent with a functional switch in moDCs in acute COVID-19 away from antigen presentation and toward neutrophil recruitment.

*C1Q*^hi^ macrophage representation in NALT remained stable in health, acute and convalescent COVID-19, but *FOLR2*^+^ macrophages significantly increased in convalescent COVID-19 (Fig. 3b). Spatially, macrophages were predominantly located in the T cell zone, including the FOLRB^+^ subset (Fig. 3f and Extended Data Fig. 4b). These *FOLR2*^+^ macrophages also showed temporal changes in their transcriptional program between acute and convalescent COVID-19, demonstrating enrichment of IL-10- and IL-4-stimulated macrophage gene signatures^28^ in convalescent disease (Fig. 3g and Extended Data Fig. 4c). EMT pathway genes highly expressed in *FOLR2*^+^ macrophages included *COL6A* (Fig. 3h), an important structural component in many tissues^29^. These *FOLR2*^+^ macrophages also expressed the tolerogenic receptor *LILRB4*, matrix metalloproteinases and *CCL18* (Fig. 3h), a profibrotic cytokine associated with the differentiation of ‘M2’-like macrophages^30^. Flow cytometric analysis confirmed increased expression of CD206, a marker of M2 macrophages, in nasal CD14^+^ cells in convalescent COVID-19 (Extended Data Fig. 4d).

Altogether, our analysis suggests that NALT myeloid cells play differing roles during the course of SARS-CoV-2 infection; infiltrating monocytes and moDCs produce self- and neutrophil-recruiting chemokines to set up a proinflammatory positive feedback loop in acute COVID-19 and localize to the periphery of NALT, forming a defensive shield. Meanwhile, NALT macrophages reside predominantly within the T cell zone and include a *FOLR2*^+^ subset that is expanded and transcriptionally activated in convalescent COVID-19, adopting a pro-repair, anti-inflammatory transcriptome, promoting tissue integrity and ensuring that the infrastructure for the generation of local adaptive immune responses is maintained.

### Clonally expanded NALT CD8 T_RM_ cells show prolonged activation

**Fig. 4:**
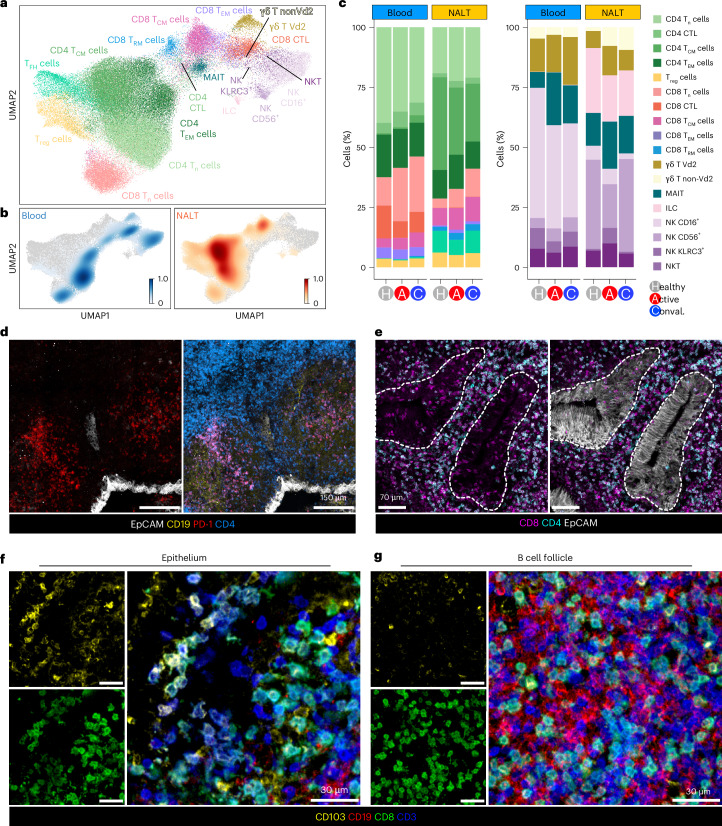
NALT CD8 T_RM_ cells exhibit clonal expansion and prolonged activation. **a**, UMAP of T cells and innate lymphocytes from participants with COVID-19 and healthy controls, reclustered in isolation, with assigned subset cell-type cluster labels shown. **b**, Scanpy embedding density (Gaussian kernel estimation) UMAP of T cells and innate lymphocytes showing scaled density of cells by sample type (blood or nasal) across the UMAP where 1 is a high density of cells of the indicated sample type. **c**, Stacked bar charts showing proportional representation of CD4 and CD8 T cells (left, paired bar chart) and innate lymphocytes (right, paired bar chart) divided by sample type (blood or NALT) and disease status. **d**, Representative confocal imaging of subepithelial B cell follicles on a section of NALT from a convalescent COVID-19 participant (*n* = 1). **e**, Representative confocal imaging of section of NALT from a convalescent COVID-19 participant (*n* = 1). **f**,**g**, Representative confocal imaging of the epithelium (**f**) and a B cell follicle (**g**) on a section of NALT from a convalescent COVID-19 participant. The arrows indicate co-expression of CD8 and CD103 (*n* = 1).

**Extended Data Fig. 5:**
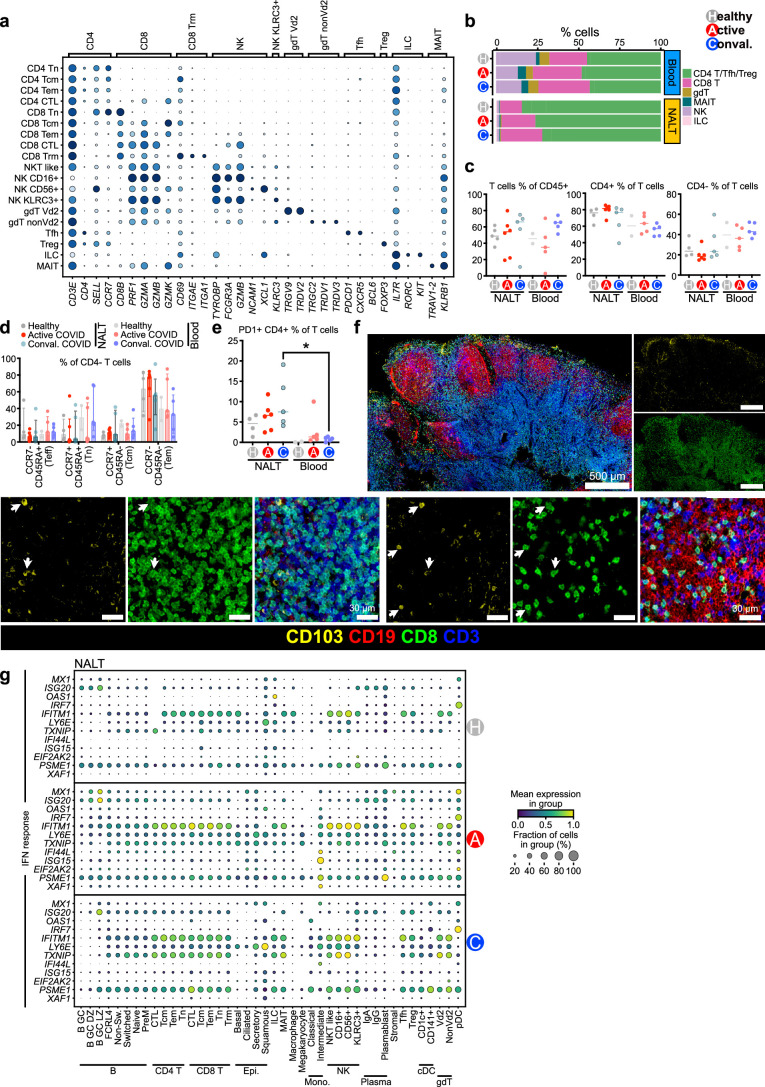
Temporal characterization of T cells and innate-like lymphocytes. **a**, Expression of canonical markers by assigned nasal T/innate lymphocyte subset cell label. Vertical axis shows assigned broad cell labels, with genes grouped by canonical expression groups on horizontal axis. Size of point indicates fraction of cells in each group expressing corresponding gene, colour of point indicates scaled mean expression of corresponding gene in each named cell type group. **b**, Stacked barcharts showing combined proportional representation of CD4 T cells, CD8 T cells and innate lymphocytes, grouped into broad cell type categories and split by sample type and disease status. **c**, Grouped scatter plot of T cell proportions, as determined by flow cytometry, split by disease type and sample type. Left, percentage of T cells in live CD45+ cells; center and right, percentage of CD4+ (centre) and CD4- (right) T cells. Each point represents an individual subject sample. Lines represent group median value. **d**, Grouped scatter and bar plot showing percentage T cell subsets based on CCR7 and CD45RA expression assessed by flow cytometry. Each point represents an individual subject sample. Bars represent median values, with lines showing interquartile range. Teff, T effector; Tn, T naïve; Tcm, T central memory; Tem, T effector memory. **e**, Grouped scatter plot showing percentage of CD4 + PD1 + T cells by tissue and condition. Each point represents an individual subject sample. Lines represent group median value. *, adjusted p < 0.05 (Šidák multiple comparisons test). **f**, Representative confocal imaging of section of NALT from a convalescent COVID-19 subject with higher magnification shown below. Related to Fig. 4f, g. n = 1. **g**, Scaled expression of selected leading edge genes in NALT cell types that were enriched in Hallmark Interferon Alpha Response and Hallmark Interferon Gamma Response (IFN Response) gene sets following gene set enrichment analysis of differentially expressed genes in COVID-19 subjects compared with healthy controls. Size of point indicates fraction of cells in each group expressing corresponding gene, colour of point indicates scaled mean expression of corresponding gene in each named cell type group. Epi, Epithelial; Mono., Monocyte.

We next considered T cell, natural killer (NK) cell and innate-like lymphocytes in isolation and identified 19 cell types (Fig. 4a and Extended Data Fig. 5a). We found distinct clusters of naive and memory CD4 and CD8 T cells, regulatory T cells (T_reg_ cells), T_FH_ cells, innate lymphoid cells (ILCs), γδ T cells, mucosal-associated invariant T cells (MAIT cells) and three subsets of NK cells marked by expression of CD16, CD56 and/or KLRC3 (Fig. 4a and Extended Data Fig. 5a). Overall, CD4 T cells were enriched in nasal samples, comprising 80% of CD3^+^ cells in NALT (Fig. 4b, Extended Data Fig. 5b, confirmed by flow cytometry, and Extended Data Fig. 5c). Tissue-resident memory (T_RM_) CD8 T cells, T_FH_ cells and ILCs were almost exclusively found in NALT samples, whereas CD16^+^ NK cells, CD8 T effector cytotoxic (CTL) and CD8 T effector memory cells were predominantly found in blood (Fig. 4b,c and Extended Data Fig. 5d,e). Spatially, programmed cell death protein 1 (PD-1)-expressing T_FH_ cells were located within B cell follicles (Fig. 4d), whereas CD8 T cells dominated within the epithelium (Fig. 4e), including CD8^+^CD103^+^ T_RM_ cells, which were also scattered in the B cell follicles and interfollicular regions (Fig. 4f,g and Extended Data Fig. 5f). In COVID-19, there was an expansion of NALT CD8 T cells compared with healthy controls, particularly naive (T_n_), central memory (T_CM_) and T_RM_ cells (Fig. 4c). The last is consistent with studies of T_RM_ cells in murine lymph nodes, demonstrating that, although most tissue CD8 T_RM_ cells remain indefinitely resident, a small number of emigrants re-locate to local draining lymph nodes, becoming broadly distributed to enable rapid expansion and defense on local rechallenge^31^.

**Fig. 5:**
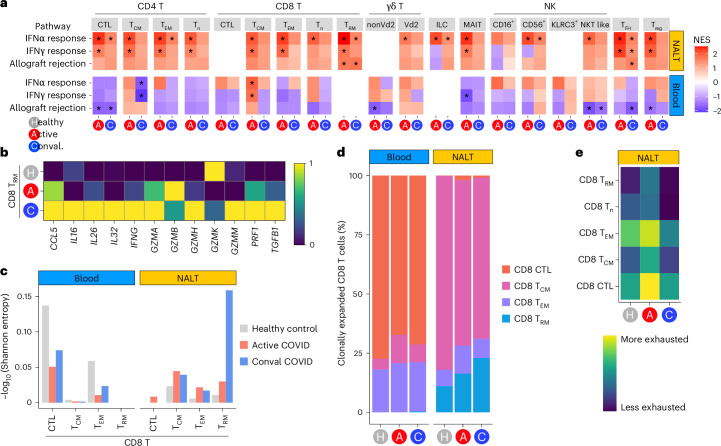
NALT CD8 T_RM_ cells exhibit clonal expansion and prolonged activation. **a**, Heatmap of a DEG enrichment of selected GSEA Hallmark gene sets in T cell and innate lymphocyte subsets by COVID status against healthy control samples. The color of the tile shows the NES. Red shows increased gene-set enrichment and blue decreased gene-set enrichment relative to healthy controls. ^*^Statistical significance *P* < 0.05 and Benjamini–Hochberg *P*_adj_ < 0.1. **b**, Scaled heatmap of mean NALT CD8 T_RM_ lymphocyte (CD8 T_RH_ cell) gene expression by disease group, showing selected gene transcripts highly expressed in CD8 T_RM_ cells in active and convalescent COVID-19 participants compared with healthy controls. **c**, TCR clonality assessment in CD8 T cell subtypes. CD8 T cell TCR diversity is indicated by a Shannon entropy index and shown by cell subset and sample type, in active COVID-19 (red) and convalescent COVID-19 (blue) compared with healthy controls (gray). The Shannon entropy value has been −log_10_(transformed) so that higher values indicate lower TCR diversity or greater TCR clonality. **d**, Bar chart showing proportional representation of clonally expanded (clone size ≥2) CD8 T cell subsets in active and convalescent COVID-19 participants compared with healthy controls, as determined by single-cell TCR analysis, split by sample type. **e**, Heatmap showing expr**e**ssion (calculated as AddModuleScore) of human CD8 T cell viral ‘exhaustion’ signature^38^ in NALT CD8 T cell subsets.

**Extended Data Fig. 6:**
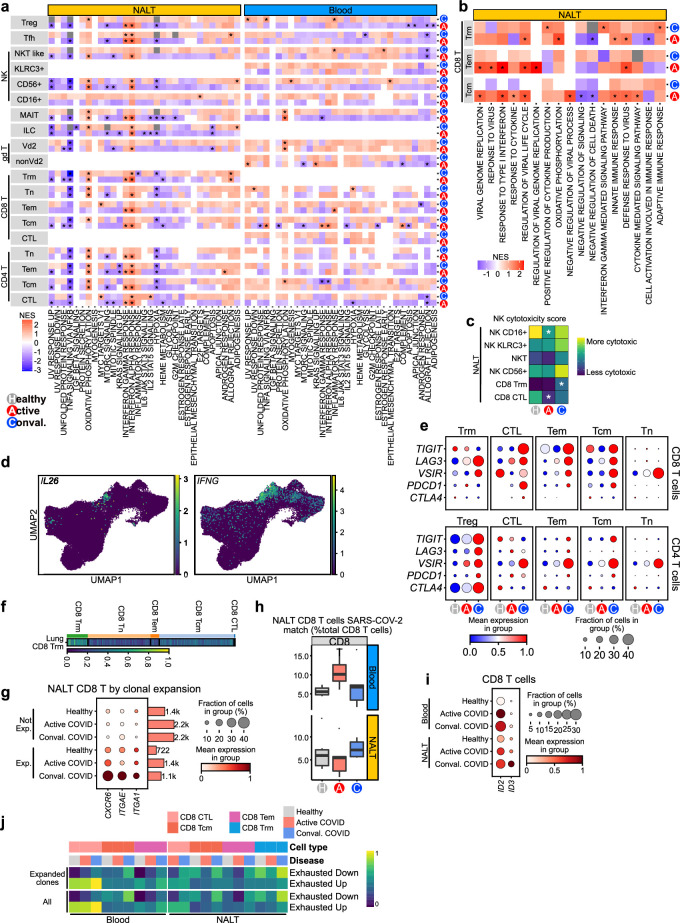
Clonally expanded NALT CD8 Trm cells show prolonged activation. **a**, Heatmap showing all all significantly enriched Hallmark pathways in T/innate lymphocyte cell types following gene set enrichment analysis of differentially expressed genes between COVID-19 disease groups and healthy control subjects. Only pathways containing at least one significant enrichment (p < 0.05 and Benjamini-Hochberg adjusted p value < 0.1) in T cell types are shown. Colour indicates normalised enrichment score (NES), with red indicating greater pathway enrichment in disease group compared with healthy controls, and blue indicating increased pathway enrichment in healthy controls than disease group. Results are split by sample type (NALT/Blood). **b**, Selected significantly enriched Gene Ontology (GO) terms in NALT CD8 T cell memory subsets, following Geneset Enrichment Analysis of differentially expressed genes in COVID-19 disease states compared with healthy controls. NES, Normalised Enrichment Score, *, p value < 0.05 + Benjamini-Hochberg adjusted p value < 0.1. **c**, Heatmap showing Gene-set scoring (AddModuleScore) of KEGG Natural killer cell mediated cytotoxicity gene set in NALT CD8 T and NK cell subsets. *, p value < 0.05. **d**, UMAP showing expression of *IL26* and *IFNG* within subset re-clustered T/innate lymphocyte UMAP. Scale bars indicate level of gene expression. **e**, Dotplot showing expression of canonical T cell exhaustion marker genes in CD4 and CD8 T cell subsets. Size of point indicates fraction of cells in each group expressing corresponding gene, colour of point indicates scaled mean expression of corresponding gene in each named cell type group. **f**, Heatmap of single cell transcriptomic data showing enrichment of human lung CD8 Trm signature^6^ in CD8 T cell subsets by assigned label. **g**, Single cell gene expression dotplot of NALT CD8 T cells showing expression of canonical CD8 Trm marker genes by disease type and clonal expansion status, with expanded clones of size >=2. Bars to right of plot indicate absolute number of cells in each group. **h**, Boxplot showing GLIPH2 identified SARS-CoV-2 specific CD8 T cells, as a proportion of total CD8 T cell population by subject and sample type and plotted by disease status. SARS-COV-2 cells were identified through TCRb similarity matching with MHC-I identified SARS-COV-2 reactive T cell sequences in the ImmunoCODE^7^ and VDJdb^8^ databases. **i**, Single cell gene expression dotplot of all CD8 T cells showing selected short- and long-term memory associated genes by disease type and sample type location. **j**, Heatmap showing enrichment of Human HIV exhaustion signatures^5^ split by cell type, sample type, disease type and clonal expansion (TCR clone size ≥2) status. Exhausted Up indicates genes upregulated in exhausted vs non-exhausted CD8 T cells and Exhausted Down indicated genes downregulated in exhausted vs non-exhausted CD8 T cells. Top two rows indicate cell type and disease status for each column.

As with myeloid cells, nasal T cell or innate lymphocyte subsets demonstrated greater infection-associated transcriptional changes compared with their circulating counterparts in acute COVID-19, particularly ‘INFα response’ and ‘INFγ response’ gene sets (Fig. 5a and Extended Data Fig. 5g). In convalescent COVID-19, the INFα response gene-set expression returned to healthy control levels in all NALT CD8 T cell subsets except for CD8 T_RM_ cells (Fig. 5a and Extended Data Fig. 6a). Indeed, CD8 T_RM_ cells showed evidence of ongoing activation in convalescent participants with significant enrichment of several immune-relevant gene sets (Fig. 5a and Extended Data Fig. 6b), including cytotoxicity-effector programs, with *IFNG* and *IL26* among notable leading-edge genes (Fig. 5b and Extended Data Fig. 6c,d). Of relevance, IL-26 binds to DNA released from damaged cells to promote damage-associated inflammation^32^ and may also act directly on epithelial cells, promoting CXCL8 production^33^. In convalescence, NALT T_RM_ cells also expressed tissue repair-associated molecules including *TGFB1* and *IL32* (Fig. 5b). Single-cell T cell receptor (TCR) analysis showed the lowest levels of diversity in the nasal CD8 T_RM_ cell population in convalescent COVID, as measured by Shannon entropy (Fig. 5c), suggestive of clonal expansion. Indeed, comparing healthy control, acute and convalescent COVID-19, a progressive increase in clonally expanded T cells (defined as TCR clonotype with ≥2 cells) was uniquely detectable in T_RM_ cells among nasal CD8 T cells (Fig. 5d and Extended Data Fig. 6e,f). Expression of canonical CD8 T_RM_ cell transcripts was also restricted to the clonally expanded population (Extended Data Fig. 6g). Using 44,139 unique major histocompatibility complex (MHC)-I-matched SARS-COV-2-specific TCR CDR3 regions from a combination of the ImmunoCODE^34^ and VDJdb^35^ databases, we used GLIPH2 (ref. ^36^) as an in silico method to identify SARS-CoV-2-specific CD8 T cells through CDR3 sequence matching. This indicated an increase in virus-specific CD8 T cells in peripheral blood in acute COVID samples, but a later expansion in convalescent NALT CD8 T_RM_ cells (Extended Data Fig. 6h). In murine models, expression of ID2 and ID3 has been linked to the establishment of short- and long-term memory CD8 T cells, respectively^37^. CD8 T cells in both NALT and peripheral blood upregulated ID2 in acute and convalescent COVID-19, but upregulation of the long-term memory marker ID3 was significant only in convalescent patients (Extended Data Fig. 6i).

Persistent CD8 T cell activation can lead to exhaustion. Overall, exhaustion-associated genes^38^ and inhibitory molecules, such as *TIGIT*, *LAG3*, *VSIR* and *PDCD1* (encoding PD-1), were upregulated in convalescent compared with acute COVID-19 (Fig. 5e and Extended Data Fig. 6e,j). In the present study, CD8 T_RM_ cells again differed from other cytotoxic nasal CD8 T cell subsets, with less enrichment of exhaustion gene-set enrichment and lower expression of *TIGIT and PDCD1* (Fig. 5e and Extended Data Fig. 6e).

Together, our data reveal marked induction of IFN-induced antiviral programs in NALT T cells in active COVID-19 infection that rapidly subsides in convalescence. The exception to this was CD8 T_RM_ cells, which alone showed clonal expansion and evidence of ongoing cytotoxic activity in convalescence, as well as production of IFNγ, antimicrobial peptides and pro-repair molecules. Remarkably, despite this persistent activation, T_RM_ cells had reduced transcriptional evidence of exhaustion compared with other CD8 T cell subsets. Although T_RM_ cells have previously been described in human secondary lymphoid organs^39^, our study provides a unique insight into how NALT T_RM_ cells respond during viral challenge, suggesting that they play parallel roles in defense, the coordination or recruitment of other T cell subsets and tissue repair.

### Antiviral GC responses in SARS-CoV-2 infection

**Fig. 6:**
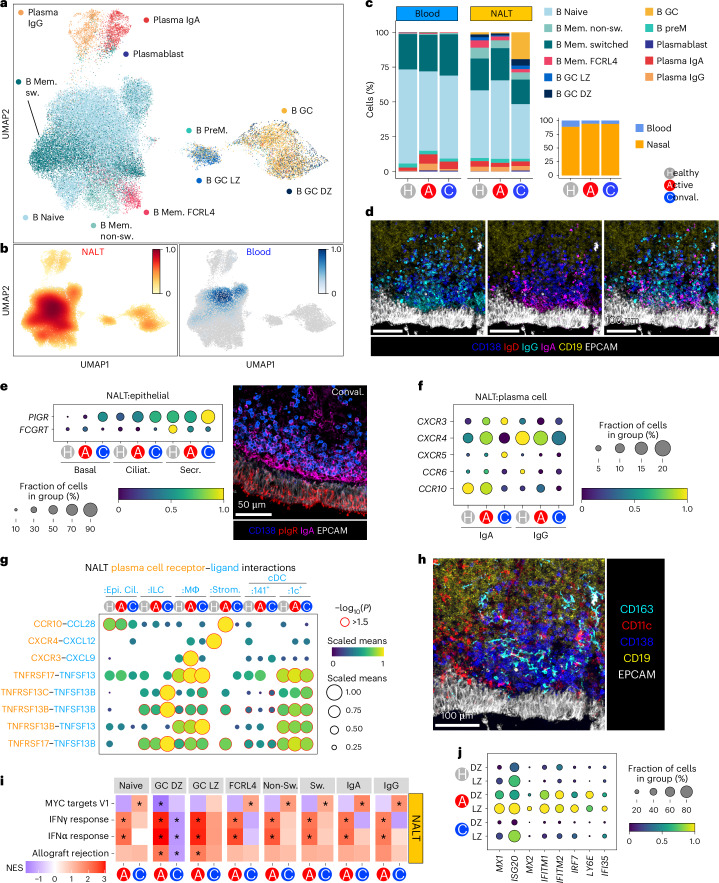
GC and PC expansion in NALT after COVID infection. **a**, UMAP of B cells and PCs from patients with COVID-19 and healthy controls, reclustered in isolation, with assigned subset cell-type cluster labels shown. **b**, Scanpy embedding density (Gaussian kernel estimation) UMAP of B cells and PCs showing scaled density of cells by sample type. NALT embedding density is shown in orange and blood embedding density in blue. **c**, Stacked bar chart showing proportional representation of B cells and PCs in blood and NALT samples by disease status. The nested bar chart on the right shows the proportional split of B cells and PCs by disease type and sample type. Mem., memory; non-sw., nonswitched; GC, germinal center; LZ, light zone; DZ, dark zone; preM, prememory. **d**, Representative confocal imaging of subepithelial space on a section from a convalescent COVID-19 participant (*n* = 1). **e**, Dotplot showing PIGR and FCGRT expression in epithelial cell subset populations in NALT by disease state (left) and representative confocal imaging of epithelium on a section from a convalescent COVID-19 participant (right) (*n* = 1). Ciliat., ciliated; Secr., secretory. **f**, Dotplot showing selected chemokine receptor expression in PC populations in NALT, split by isotype. **g**, Dotplot showing CellPhoneDB cell–cell interaction prediction analysis for NALT PCs and ciliated epithelial cells, ILCs, macrophages, cDCs and stromal cells. The size and color of the dot indicate scaled mean, where the mean value refers to the total mean of the individual partner average expression values in the corresponding interacting pairs of cell types. The red dot border highlight indicates significance (−log_10_(*P*) > 1.5). The *P* values were calculated in the CellPhoneDB package using 1,000 permutations. **h**, Representative confocal imaging of the subepithelial space on a section from a convalescent COVID-19 patient (*n* = 1). **i**, Heatmap of DEG enrichment of GSEA Hallmark gene sets in B cell and PC subsets by COVID status against healthy control samples. The color of the tile shows the NES (red is increased gene-set enrichment and blue decreased gene-set enrichment relative to healthy controls), ^*^Statistical significance *P* < 0.05 and *P*_adj_ < 0.1. NESs were calculated in the fgsea package with *P*-value estimation using an adaptive, multi-level, split Monte-Carlo scheme. **j**, Dotplot showing expression of selected, highly expressed, type I IFN response genes in B cell subsets in NALT. DZ, dark zone; LZ, light zone. In **e**, **f** and **j**, the size of the point indicates the fraction of cells in each group expressing the corresponding gene and the color of the point indicates the scaled mean expression of the corresponding gene in each named cell-type group.

**Extended Data Fig. 7:**
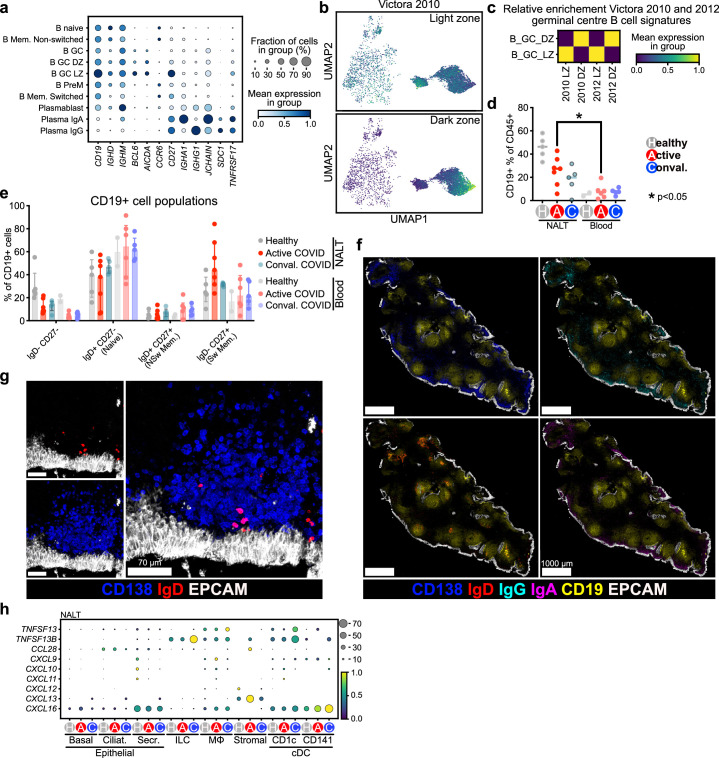
Local anti-viral germinal centre response in NALT. **a**, Expression of canonical markers by assigned B lymphocyte subset cell label. Vertical axis shows assigned broad cell labels, with genes grouped by canonical expression groups on horizontal axis. Size of point indicates fraction of cells in each group expressing corresponding gene, colour of point indicates scaled mean expression of corresponding gene in each named cell type group. **b**, UMAP showing gene set enrichment score (AddModuleScore) of experimentally derived Germinal centre B cell signatures^9^ in germinal centre B cell clusters. **c**, Single cell gene expression matrixplot of germinal centre B cell light zone (B_GC_LZ) and dark zone (B_GC_DZ) labelled cells, showing relative enrichment of experimentally derived mouse (2010^9^) and human (2012^10^) light zone (LZ) and dark zone (DZ) germinal cell B cell specific cell signatures. **d**, Grouped scatter plot showing percentage of CD19+ cells in live CD45+ cells, as determined by flow cytometry. Each point represents an individual subject sample. Lines show group median value. Data is divided by disease and sample type. *, p < 0.05 (Wilcoxon test). **e**, Grouped scatter and bar plot of CD19+ cell subpopulation proportions by IgD and CD27 expression, as determined by flow cytometry. Each point represents an individual subject sample. Bars represent median values, with lines showing interquartile range. NSw Mem, Non-switched memory; Sw. Mem, Switched memory. **f-g**, Representative confocal imaging of section of NALT from a convalescent COVID-19 subject (**f**) with higher magnification for three of the markers (**g**). n = 1. **h**, Dotplot showing selected chemokine expression across NALT Epithelial cells, ILCs, Macrophages (MΦ), Stromal cells and conventional dendritic cells. Size of point indicates fraction of cells in each group expressing corresponding gene, colour of point indicates scaled mean expression of corresponding gene in each named cell type group.

In the B cell compartment, we identified naive, nonswitched and switched memory, FCRL4^+^ mucosa-associated memory B cells, immunoglobulin (Ig)A/IgG plasma cells (PCs) and early plasmablasts, as well as distinct clusters of GC-associated populations, including dark zone, light zone and memory precursors^4,40,41^ (Fig. 6a and Extended Data Fig. 7a–c). Cells annotated as B GCs showed expression of canonical GC markers (*AICDA* and *BCL6*), but failed to enrich for either a light or dark zone signature specifically.

Most B cells profiled originated from nasal samples (Fig. 6b,c), confirmed by flow cytometry (Extended Data Fig. 7d,e). The nasal B cell compartment was proportionally expanded in both acute and convalescent COVID-19 compared with healthy controls, with a prominent increase in GC populations in convalescent samples (Fig. 6b). FCRL4^+^ memory cells decreased in COVID-19 and there was also a proportional reduction in nasal switched memory B cells in convalescence compared with acute disease (Fig. 6b), confirmed by flow cytometry (Extended Data Fig. 7e).

Spatially, PCs were layered along the entire subepithelium in convalescence, with many IgG^+^ and IgA^+^ PCs co-localizing here, along with occasional IgD^+^ PCs (Fig. 6d and Extended Data Fig. 7f,g), the latter previously described in tonsils and the nasopharynx^42^. Expression of the polyclonal Ig receptor (PIGR), required for transepithelial shuttling of IgA^43^, was evident in NALT epithelium and increased in convalescent COVID-19 (Fig. 6e), augmenting the capacity to transport IgA generated by adjacent PCs into the nasal space. Transepithelial transport of IgG requires the neonatal Fc receptor (FcRn)^44^. *FCGRT* transcripts were detectable in nasal epithelial cells but did not show an increased post-COVID-19 infection and, indeed, even decreased (Fig. 6e), potentially indicating that IgG produced in NALT may bolster systemic rather than nasal IgG. Most IgG^+^ PCs, as well as many IgA^+^ PCs, expressed *CXCR4* (Fig. 6f), enabling localization to a *CXCL12*-expressing niche. The remaining IgA^+^ PCs expressed *CCR10 and CXCR3* (Fig. 6f), with NALT stromal cells the only detectable source of *CCL28* (the ligand for CCR10), but myeloid cells the major source of the CXCR3 ligands *CXCL9*, *CXCL10* and *CXCL11* (Fig. 6g and Extended Data Fig. 7h). Indeed, macrophages and DCs also expressed *TNFSF13* and *TNSF13B* (encoding APRIL and BAFF, respectively; Fig. 6g and Extended Data Fig. 7h), survival factors for PCs^45^, and co-localized with PC clusters (Fig. 6h).

**Extended Data Fig. 8:**
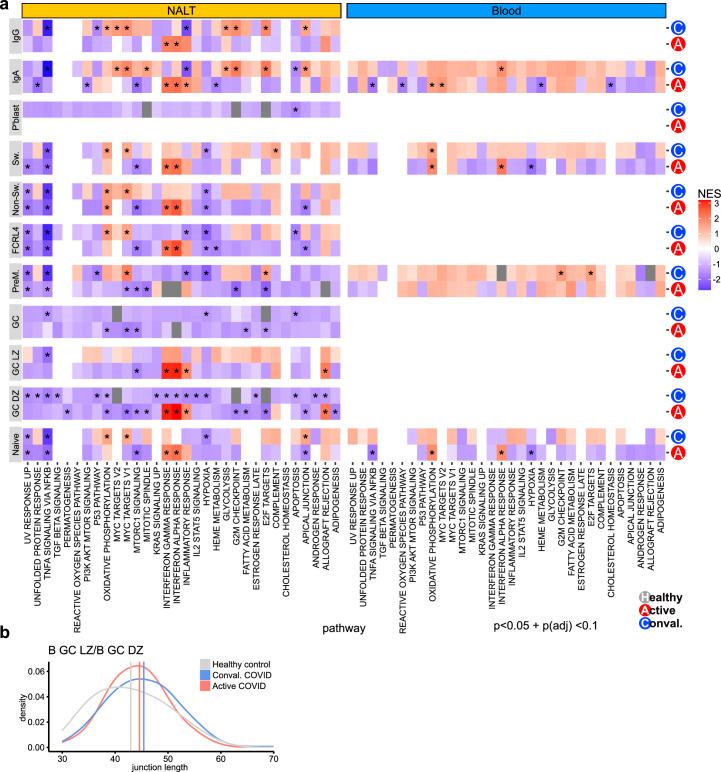
V(D)J rearrangement and selection in NALT. **a**, Heatmap showing all significantly enriched Hallmark pathways in B lymphocyte/plasma cell types following gene set enrichment analysis of differentially expressed genes between COVID-19 disease groups and healthy control subjects. Only pathways containing at least one significant enrichment (p < 0.05 and Benjamini-Hochberg adjusted p value < 0.1) in B cell types are shown. Colour indicates normalised enrichment score (NES), with red indicating greater pathway enrichment in disease group compared with healthy controls, and blue indicating increased pathway enrichment in healthy controls than disease group. Results are split by sample type (NALT/Blood). **b**, Density plot of BCR junction length in light zone and dark zone germinal centre B cells, split by condition. Vertical lines indicate mean junction length for each disease group.

Nasal B and plasmablast cells showed induction of IFNα response pathway genes in acute COVID-19 (Fig. 6i and Extended Data Fig. 8a). Notably, GC B cells showed substantial expression of several type I IFN-dependent antiviral genes, including *MX1*, *ISG15*, *IRF7* and *IFITM1/2* (Fig. 6j). MX1 directly inhibits viral ribonucleoprotein complexes and has been implicated in SARS-CoV-2 defense^46^ and its expression by GC B cells indicates that these specialized adaptive immune cells retain cell-autonomous antiviral capacity while simultaneously differentiating to produce progeny with class-switched, somatically mutated B cell receptors (BCRs). Indeed, among immune cells, in acute COVID-19, light zone B cells showed the highest expression of IFN-induced protein 20 (*ISG20*), an RNA exonuclease with broad antiviral properties^47^ (Fig. 6j).

**Fig. 7:**
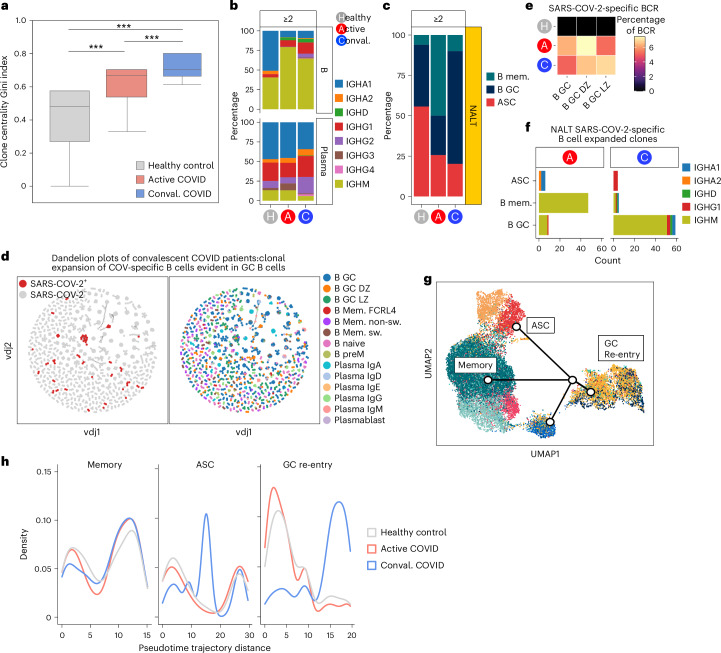
Type I IFN response and GC cell fate and progression. **a**, Box plot of NALT BCR Gini centrality index, as a measure of B cell clonality, split by disease state. Gini index values are on a scale from 0 to 1, where 1 indicates a monoclonal, highly mutated clonal response and 0, conversely, a polyclonal and unmutated response. Data are shown as box plots (median, box as 25th and 75th percentiles and whiskers as 1.5× the interquartile range). ^***^P < 0.001, two-sided Wilcoxon’s rank-sum test (*n* = 10 healthy, *n* = 8 active, *n* = 5 convalescent). **b**, Bar chart showing proportional representation of Ig heavy chain isotypes in expanded NALT BCR clones (clone size ≥2), split by cell type and disease state. **c**, Bar chart showing proportional representation of memory, GC cells and ASCs in expanded NALT BCR clones. **d**, Single-cell BCR network plots for convalescent COVID-19 participants. Each circle or node corresponds to a single B cell with a corresponding set of BCR(s). Each clonotype is presented as a minimally connected graph with edge widths scaled to 1 per *d* + 1 for edge weight *d*, where *d* corresponds to the total (Levenshtein) edit distance of BCRs between two cells. The left-hand plot shows SARS-COV-2-specific clones (red) in the context of all B cell clones and the right-hand plot the assigned cell-type labels derived from gene expression data. **e**, Heatmap showing SARS-COV-2-specific BCRs as a percentage of total sequenced single-cell BCRs in GC B cells, by condition. **f**, Stacked bar chart showing counts of expanded (≥2) SARS-COV-2-specific B cell clones in NALT, by disease state, cell type and isotype. The *x* axis shows the cell type and the *y* axis the count; the color shows the isotype. B mem, memory B cell. **g**, B lineage cell Slingshot pseudotime trajectories, plotted in UMAP (corresponding to Fig. 6a). The start point is pinned on to B_GZ_LZ (light zone GC, royal blue). **h**, Cell density map along pseudotime trajectory distance for memory, GC re-entry and ASC lineages, split by disease state. The *x* axis indicates the pseudotime trajectory distance and the *y* axis the density of cells.

BCR analysis showed an increase in CDR3 junctional length in GC B cell populations in convalescent COVID-19 compared with other groups, suggesting increased V(D)J rearrangement and selection (Extended Data Fig. 8b). The Gini index (a measure of clonal selection) was also significantly increased in convalescent COVID-19 (Fig. 7a). Analysis of class switching in expanded clones (more than two) showed a marked expansion of IgM^+^ clones (predominantly nonswitched memory B cell clones) and IgG1 and IgG2 clones (predominantly GC B cells in active COVID-19, with IgG clones more numerous in convalescence) (Fig. 7b). Thus, class switching to IgG rather than IgA appears to be the favored local mucosal response to SARS-CoV-2 infection. In acute COVID, we found an expansion of memory B cell clones, with expanded GC clones more prominent in convalescence (Fig. 7c).

**Extended Data Fig. 9:**
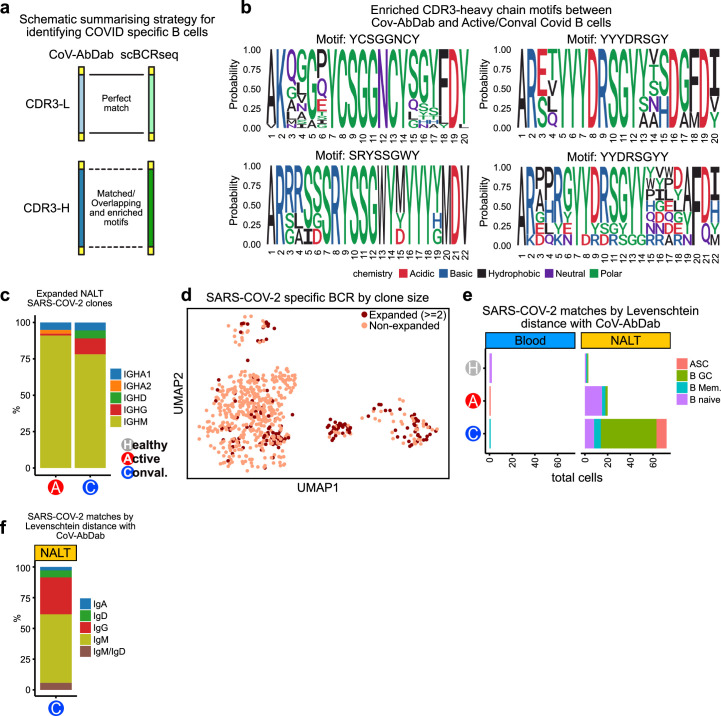
Local production of anti-SARS-COV-2 antibodies in NALT following infection. **a**, Schematic summarising the strategy for identifying SARS-COV-2 specific B cells from the single cell CDR3-L and CDR3-H sequences shared with CoV-AbDab. **b**, Enriched CD3-heavy chain motifs between Cov-AbDab and B cells from Active/Conval COVID-19 subjects. **c**, Stacked bar plot showing proportional split by isotype of expanded (clone size >=2) SARS-COV-2 specific B cell clones by disease state. **d**, UMAP showing SARS-COV-2 specific B cell clones plotted using co-ordinates from B/Plasma UMAP seen in Fig. 6a. Cells forming part of expanded BCR clones (clone size >=2) are shown in dark red, with clonally unexpanded SARS-COV-2 specific B cells shown in pink. **e**, Stacked barplot showing absolute numbers of SARS-COV-2 specific B cells identified by both Gliph2 and Levenschtein distance heavy chain matching with CDR3 sequences from Cov-AbDab11 presented by sample type, disease type and assigned cell type label. **f**, Stacked barplot showing proportional representation of isotypes in SARS-COV-2 specific B cells from Convalescent COVID-19 subecjts, identified by both Gliph2 and Levenschtein distance heavy chain matching with CDR3 sequences from Cov-AbDab^11^.

Next, we sought to identify SARS-CoV-2-specific BCRs, comparing our single-cell BCR data to a reference COVID antibody database (CoV-AbDab)^48^. First, using GLIPH2 (Grouping of Lymphocyte Interactions by Paratope Hotspots v.2) to perform heavy chain matching with CoV-AbDab, SARS-CoV-2-binding BCR sequences were defined as cells with an identical CDR3-L sequence and a paired CDR3-H with significant motif similarity to an antibody within the CoV-AbDab (Extended Data Fig. 9a,b). Putative SARS-CoV-2-binding BCRs (*n* = 768) comprised around 5% of GC B cell BCRs in patients with COVID-19, but were not detectable in control GC B cell BCRs (Fig. 7d,e). Clonal expansion of SARS-CoV-2-specific B cells was also principally evident among GC B cells in convalescent COVID-19 participants (Fig. 7f and Extended Data Fig. 9c,d), supporting the conclusion that this lymphoid tissue generates early local antiviral antibodies in COVID-19. As GLIPH2 is typically used for TCR matching, we also matched SARS-CoV-2-specific B cells identified by GLIPH2 with SARS-CoV-2-specific cells identified by Levenshtein distance similarity to CoV-AbDab BCRs. Although this identified a smaller number of SARS-CoV-2-specific BCRs, the pattern of expansion of SARS-COV-2-specific B cells mirrored the results generated by GLIPH2 (Extended Data Fig. 9e,f).

**Extended Data Fig. 10:**
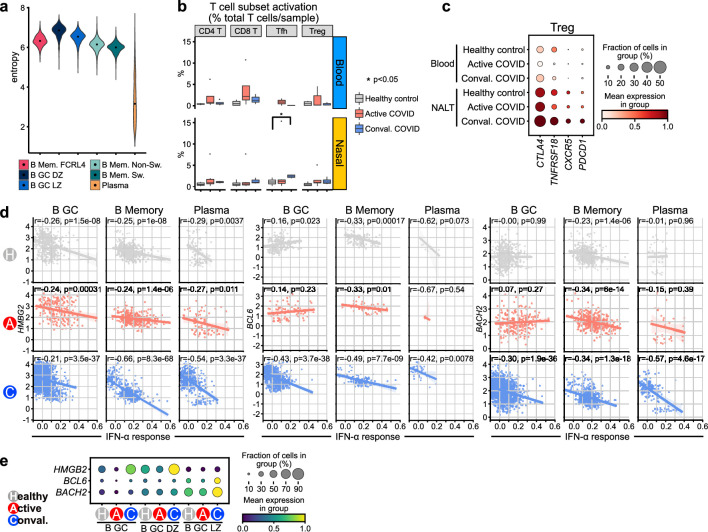
Type I interferon may inhibit the progression of the GC reaction during infection. **a**, Violin plot of per-cell transcriptomic entropy for B cells by subset cell type label. **b**, Boxplot of enrichment of a T cell activation signature induced by controlled SARS-COV-2 infection^12^ in T cells from NALT and peripheral blood by disease status. Two sided significance is indicated by *. **c**, Single cell gene expression dotplot showing expression of selected inhibitory and lymphoid homing receptors in T regulatory cells by sample type and disease status. **d**, Extended figures from Fig. 6k, showing plots split by cell type and disease type for interferon alpha response scores plotted against expression of HMGB2 (upper left), BCL6 (upper right), and BACH2 (lower left). Linear regression line is shown with a shaded area representing the 95% confidence interval. r, Pearson correlation statistic; p, p-value. *, p < 0.05; **, p < 0.01; ***, p < 0.001; ns, p > 0.05. **e**, Dotplot showing expression of genes shown in Fig. 8d in NALT germinal centre B cells, split by cell type and disease type. Size of point indicates fraction of cells in each group expressing corresponding gene, colour of point indicates scaled mean expression of corresponding gene in each cell type and disease group.

The cues that determine which cellular fate a GC B cell adopts and what signals initiate GC exit to a memory B cell or PC fate in humans remain unclear. Murine studies suggest that this decision is influenced by a combination of BCR antigen affinity, local cytokine environment and the provision of co-receptor signals by T_FH_ cells. A temporal model has been proposed, where the GC response undergoes a switch in its output as it matures, with memory B cells preferred at early time points and PCs later^49^. Temporal studies of GC progression in lymphoid organs in humans are lacking, so, to investigate this, we used Slingshot, pseudotime, cell-fate trajectory analysis^50^. To specify the root of this trajectory analysis, we used cellular entropy, a marker of transcriptional diversity that has been proposed as inversely proportional to cellular differentiation^51^. After dark zone GC cells, which are known to increase their genetic diversity through somatic hypermutation, this identified the B GC light zone cluster (B_GC_LZ) as the least differentiated (Extended Data Fig. 10a). Pseudotime trajectory analysis rooted in the light zone GC cluster revealed three distinct cell trajectories, one terminating in the antibody-secreting cell (ASC) cluster, the second in the switched memory B cell cluster and the third in the GC dark zone population, which we termed a ‘GC re-entry’ fate (Fig. 7g). When comparing disease groups and control, we found a paucity of cells in the terminal stages of the GC re-entry trajectory in acute COVID-19, in contrast to convalescent COVID-19, where many cells were evident in the late GC re-entry trajectory. Cells from convalescent COVID-19 patients also showed an early peak in density in the ASC trajectory compared with acute COVID-19 and controls (Fig. 7h). Taken together with our analysis of expanded clones, our pseudotime data are consistent with a temporal model of GC progression.

**Fig. 8:**
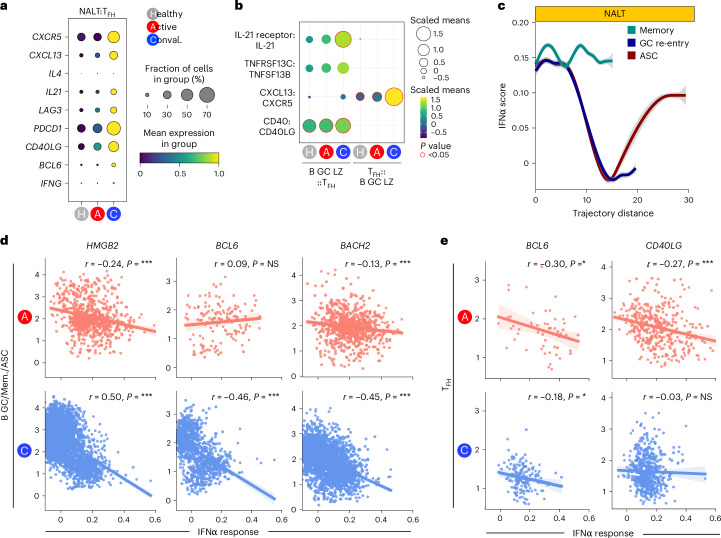
Type I IFN response and GC cell fate and progression. **a**, Dotplot showing gene expression in NALT T_FH_ cells, by disease state, of selected core genes associated with T_FH_ cell polarization and function. The size of the point indicates the fraction of cells in each group expressing the corresponding gene and the color of the point the scaled mean expression of the corresponding gene in each disease group. **b**, Dotplot showing CellPhoneDB cell–cell interaction prediction analysis for NALT light zone GC B cells (B GC LZ) and T_FH_ cells. The size and color of the dot indicate the scaled mean, where the mean value refers to the total mean of the individual partner average expression values in the corresponding interacting pairs of cell types. The red dot border highlight indicates the significance (−log_10_(*P*) > 1.5). **c**, Line graph of type I IFN response gene-set (GSEA Hallmark IFNα response) AddModuleScore in memory, GC and antibody-secreting B lineage cells along a pseudotime trajectory distance for memory, GC re-entry and ASC trajectories (trajectories shown in Fig. 6d). Memory is teal, GC re-entry dark blue and ASC red. **d**, Scatter plot with linear regression line plotting expression of type IFN response (GSEA Hallmark IFNα response) AddModuleScore against HMGB2, BCL6 and BACH2 expression in GC and memory B cells and PCs. The linear regression line is shown with a shaded area representing the 95% CI. ^*^P < 0.05, ^**^P < 0.01, ^***^P < 0.001, NS (nonsignificant) *P* > 0.05. **e**, Scatter plot with linear regression line plotting expr**e**ssion of the type IFN response (GSEA Hallmark IFNα response) AddModuleScore against *BCL6* and *CD40L* in T_FH_ cells. The linear regression line is shown with a shaded area representing the 95% CI. ^*^P < 0.05, ^**^P < 0.01, ^***^P < 0.001, NS *P* > 0.05.

Murine studies suggest that the switch in GC output over time may be mediated by changes in the transcriptional characteristics of T_FH_ cells, with the ratio IL-21:IL-4 decreasing over time, and a switch toward IL-4 production. In our dataset, T_FH_ cells showed increased expression of *IL21* in convalescent COVID-19 compared with acute disease and control samples, with little *IL4* detectable (Fig. 8a). T_FH_ cell–light zone GC B cell interaction prediction using CellPhoneDB also showed a temporal increase in several interactions that would promote GC progression, including *CXCL13* *CXCR5*, *TNFRSF13C*–*TNFSF13B*, *IL21R*–*IL21* and *CD40*–*CD40L* in convalescent COVID (Fig. 8b). Using cell2TCR^52^, we matched T cell subsets in the NALT to an experimentally derived T cell activation signature generated from a SARS-CoV-2 human infection model^52^, and used this to plot T cell subset activation profiles in NALT over the course of infection. This showed a significant increase in T_FH_ and follicular regulatory T (T_FR_) cell activation in convalescent disease, the latter T cell expressing increased levels of *TNFRSF18* (GITR), CTLA-4 and PD-1 (Extended Data Fig. 10c).

We hypothesized that, during viral infection, type I IFNs may provide an environmental cue that influences GC output. Assessment of gene expression showed persistent induction of type I IFN response genes in cells across the memory B cell trajectory, in contrast to cells progressing along a GC re-entry or ASC trajectory (Fig. 8c). It is interesting that, in GC B cells, there was a negative correlation between type I IFN response genes and *HMGB2* and *BACH2* in SARS-CoV-2 infection, particularly in convalescent disease (Fig. 8d and Extended Data Fig. 10d,e). In NALT T_FH_ cells, type I IFN response genes showed a negative correlation with *BCL6* in COVID-19 (Fig. 8e). Altogether, this is consistent with the conclusion that type 1 IFN may inhibit the progression of the GC reaction, potentially providing an explanation as to why severe COVID-19 is associated with limited GC formation.

## Discussion

Although there is macroscopic involution of the pharyngeal tonsils with age, our study showed that the postnasal space (PNS) contains lymphoid tissue in adults that is responsive to viral infection and can be sampled to profile the cellular molecular processes occurring during the generation of adaptive immune responses in living participants. We showed that these NALT biopsies can be collected in an outpatient clinic setting, providing a means of longitudinally assessing secondary lymphoid tissue in humans. NALT immune responses are particularly relevant to pathogens that enter via the nasal mucosa, including SARS-CoV-2, and to nasal vaccines^10,11^. We demonstrated that NALT profiling enables the study of cell types that are central to adaptive immunity, but not present in blood, particularly GCs and stromal cells. A recent study using PNS swabs similarly captured adenoid GC populations in the context of SARS-CoV-2 infection^53^. This methodology has the advantage of enabling longitudinal sampling but does not provide spatial information or include stromal or significant numbers of myeloid cells.

Lymphoid tissues contain several myeloid cell populations. We found that, in acute COVID-19, monocytes formed a superficial lining in NALT, expressing the antibacterial protein calprotectin^21^, as well as self- and neutrophil-recruiting chemokines. Neutrophils were also evident in this zone, some of which had histone citrullination, required for chromatin decondensation and NET formation^22^. NETs can also entrap and kill bacteria^54^, potentially acting together with monocytes to defend the underlying follicles and T cells from nasal bacteria during a period of vulnerability when overlying epithelial cells are damaged or killed by invading virus.

Murine studies show that subcapsular sinus macrophages play important roles in viral defense^8^. Furthermore, lymphatic sinus-lining macrophages produce IL-18 in response to bacterial infection, stimulating innate lymphocytes to produce IFNγ, which enhanced macrophage antibacterial function^9^. The pharyngeal tonsils do not have afferent lymphatic drainage via a subcapsular sinus, but rather antigens and pathogens come directly from the overlying nasal space. In the present study, we found that human NALT contains a dense network of macrophages, including a *FOLR2*^+^ population, which expanded in convalescent COVID-19, expressing *IL18*, and pro-repair molecules, including type VI collagen.

Murine models of upper respiratory tract influenza challenge have shown CD8 T_RM_ cell expansion in the nasal tissue^55^ and SARS-CoV-2-specific CD8 T_RM_ cells have also been described in human nasal mucosa^56^. Our study confirmed their presence in NALT and showed that, unlike other CD8 T cell subsets, T_RM_ cells clonally expand and show prolonged cytotoxic gene expression in convalescence, with little evidence of exhaustion. This is in contrast to previous reports suggesting T_RM_ cell exhaustion in active COVID-19 infection^57^. It is interesting that NALT T_RM_ cells also expressed lymphocyte-recruiting chemokines and *TGFB1*, suggesting that they play additional roles beyond immediate antiviral defense.

We found reciprocal changes in the expression of IgG and IgA transporters in NALT epithelium, which suggest an increase in IgA transport to the nasal mucosa, but that NALT IgG contributes to systemic immunity. We did not take nasal secretion or saliva samples to directly measure Ig levels; however, studies delineating secretory IgA, IgA and IgG responses in SARS-CoV-2 support this finding^58,59^.

Our longitudinal analysis of GC progression in SARS-CoV-2 infection provides unique insights into the dynamics of GC responses in humans. Although studies in mice suggest a temporal model where memory B cells emerge earlier in responses, this has been difficult to confirm in humans. We found increased representation of memory B cells in expanded clones in acute COVID-19, which decreased in convalescence, and our pseudotime analysis showed a peak in cell density in the ASC trajectory in convalescence. Together, these analyses are supportive of a temporal model of GC progression. We also found that type I IFN may provide an environmental cue that influences GC output, with memory cells showing the highest expression of type I IFN response genes throughout their trajectory.

In the present study, we showed that adults with mild COVID-19 disease do form GCs in response to SARS-CoV-2. This enabled us to probe potential mechanisms of GC collapse described in fatal disease^19^. We found that high IFN response gene expression was associated with reduced expression of *BACH2* and *BCL6* in GC B cells and reduced expression of *CD40L* in T_FH_ cells. This implies that an excess of type I IFNs, as has been described in severe disease, may inhibit the progression of the GC reaction.

A limitation to our study is that the acute and convalescent samples were not taken from the same participants and two out of eight of the acute COVID-19 patients were on corticosteroids, one on corticosteroids and baricitinib and one on corticosteroids and tocilizumab, compared with the five convalescent participants, none of whom had severe disease or were on immunomodulating medications, which may influence the acute and convalescent comparisons.

In summary, our study provides a unique insight into how nasal adaptive immune responses are generated and defended during SARS-CoV-2 infection. Beyond COVID-19, our study provides proof of principle that this sampling strategy will enable long-sought efforts to interrogate longitudinal changes in lymphoid organs in humans in disease and after therapeutic intervention.

## Methods

The list of reagents used in the present study can be found in Supplementary Table 2.

### Human participant recruitment

COVID-19 participants were recruited from a single site—Addenbrooke’s Hospital, Cambridge, UK—between May 2020 and July 2021. Healthy control individuals were recruited between August 2019 and November 2019 from Cambridge, UK. Ethical approval was given through the Cambridge Central Research Ethics Committee (reference no. 08/H0308/267, IRAS project no. 194217), administered through Cambridge University Hospitals NHS Foundation Trust. All participants provided informed written consent and, thus, patients unable to provide this, including those undergoing invasive ventilation, were excluded. The following participants were also excluded: aged <18 years, with a chronic systemic infection (for example, HIV, viral hepatitis), with a diagnosis of active malignancy, previous head and neck radiotherapy, a diagnosis of systemic autoimmune disease (for example, rheumatoid arthritis, ANCA-associated vasculitis) or a known nasal or postnasal anomaly precluding access or safe biopsy (for example, severe septal deviation, nasal vascular malformation). All active COVID-19 participants, with the exception of participant no. 1, were recruited within 7 d of their first positive swab (quantitative RT–PCR) result and within 14 d of symptom onset. Participant no. 1 was recruited on the basis of radiological changes consistent with COVID-19, in the presence of positive COVID-19 serology and the onset of symptoms consistent with COVID-19 within the past 14 d. All convalescent COVID-19 participants were recruited between 21 d and 28 d after a positive COVID-19 swab result. No COVID-19 participants had received a COVID-19 vaccine at the time of recruitment.

### Sample collection

Nasal samples were collected by an otolaryngologist under direct endoscopic visualization. Briefly, the nasal cavity was anesthetized with topical local anesthetic and epinephrine (lidocaine 5%:phenylephrine 0.5%) administered via spray and neurosurgical patties (Codman). Nasal brushings and microcurettage samples were collected from the inferior turbinate under direct vision with the aid of a Thudichum nasal speculum. The PNS was inspected transnasally with a 0° nasendoscope and one to three biopsies taken from the PNS mucosa using Tilley–Henkel, Takahashi fine-cupped or Blakesley forceps, and placed directly into Roswell Park Memorial Institute (RPMI)-1640 medium on ice. Blood samples were collected contemporaneously into sodium citrate tubes using the vacutainer system (BD)

### Sample processing to single-cell suspension

All samples were processed fresh, within 1 h of collection. PNS samples were washed in RPMI-1640 medium, diced and placed into a 15-ml Falcon tube with 5 ml of RPMI-1640 medium and a digestion solution of 62.5 μl of Liberase (stock concentration 1 mg ml^−1^; Sigma-Aldrich) and 250 μl of DNase I (stock concentration 2.5 mg ml^−1^; Roche). The sample tube was placed into a shaking incubator at 37 °C for 30 min at 220 r.p.m. The sample was then pushed through a 70-μm cell strainer (Falcon) into a 50-ml Falcon tube and washed in 50 ml of RPMI-1640 medium at 500*g* for 6 min with the brake on. The cell pellet was resuspended in 10 ml of 44% Percoll solution and centrifuged at 800*g* for 20 min and 20 °C with no brake. The supernatant was aspirated. If the cell pellet had visible blood staining, 2 ml of ACK red cell lysis buffer was added for 2 min, before quenching with 20 ml of 1× phosphate-buffered saline (PBS) and centrifuging at 500*g* and 4 °C for 6 min (brake on). After aspirating the supernatant, the cell pellet was resuspended in 100 μl of 1× PBS. Nasal brushing heads were vigorously agitated 15× in 5 ml of RMPI-1640 collection fluid. The brush head was cut off and left in the collection fluid while it was centrifuged at 500*g* for 6 min at 4 °C (brake on). The brush head and supernatant were removed and the cell pellet resuspended in 100 μl. Nasal microcurettes were agitated in 5 ml of RPMI-1640 collection fluid before centrifugation at 500*g* for 6 min (4 °C, brake on). The resulting cell pellet was resuspended in a digestion fluid of Liberase or DNase I at the same concentration as the PNS samples and placed into a shaking incubator for 20 min at 37 °C and 220 r.p.m. After digestion, samples were disassociated by pipette mixing and washed at 500*g* for 6 min (brake on) and resuspended in 100 μl of 1× PBS. Then, 18 ml of blood from the sodium citrate collection tubes was diluted 1:1 with 1× PBS and 15 ml of Histopaque 1077 (Sigma-Aldrich) was added to each of two 50-ml Falcon tubes, with the blood–PBS mixture layered on top. Each tube was centrifuged at 800*g* for 20 min at room temperature (low brake). The resulting PBMC layer was aspirated with a Pasteur pipette and washed twice in 50 ml of PBS before being resuspended in 500 μl of 1× PBS.

The samples were counted with Trypan Blue staining to assess cell counts and viability, before downstream applications.

### ScRNA-seq library generation

PNS samples were pooled with curettage and brushing cells at a ratio of 2:1:1. Where nasal curettage and/or nasal brushing cell counts were insufficient or unavailable, only PNS samples were used. Blood and nasal samples were diluted to a concentration of 2 × 10^6^ cells per ml and loaded on to a 10x Chromium controller in parallel lanes with a targeted cell recovery of 14,000 cells. Single-cell libraries + TCR and BCR VDJ libraries were created using Chromium Next GEM (Gel Beads in Emulsion) Single cell 5′ kit v.1.1 (10x Genomics), in accordance with the manufacturer’s user guide. Samples were sequenced using a Novaseq 6000 S4 system (Illumina).

### Flow cytometry

After scRNA-seq GEM creation, the remaining single-cell suspensions were diluted to a concentration of 2 × 10^6^ cells in 100 μl of 1× PBS. If this concentration was not achievable, all remaining cells were used. Cells were blocked with 5 μl of FcR blocking reagent (Miltenyi-Biotech) before undergoing surface staining with fluorophore-labeled antibodies. The antibodies used in the present study are listed in Supplementary Table 2. The samples were washed and resuspended in 1× PBS and run on a Fortessa II flow cytometer (BD). Data were gated into populations using FlowJo (Treestar/BD). All populations used in this publication were gated on a starting population of CD45^+^ live singlets. Gating strategies are available in Supplementary Fig. 1.

### Immunofluorescence microscopy

PNS biopsies were collected as described above and fixed for 15–30 min (depending on tissue size) in AntigenFix (DiaPath, cat. no. P0016), followed by 8 h in 30% sucrose in PBS before embedding in OCT (CellPath, cat. no. KMA-0100-00A). Then, 15-µm sections were mounted on Polysine slides (Thermo Fisher Scientific, cat. no. J2800AMNZ) and permeabilized and blocked in 0.1 M Tris, pH 7.4 containing 0.1% Triton (Sigma-Aldrich, cat. no. 93426-100ML), 1% normal mouse serum (Invitrogen, cat. no. 10410), 1% normal donkey serum (Abcam, cat. no. ab7475) and 1% bovine serum albumin (R&D Systems, cat. no. DY995) for 1 h at 20 °C. Samples were stained for 2 h at 20 °C (primary antibodies) or 1 h at 20 °C (secondary antibodies) in a humid chamber with the appropriate antibodies, washed 3× in PBS and mounted in Fluoromount-G (Southern Biotech, cat. no. 0100-01). Images were captured using a TCS SP8 (Leica) inverted microscope, on a ×40, 1.1 numerical aperture, water immersion objective. Raw imaging data were processed using Imaris (Bitplane/Oxford Instruments). All antibodies used in the present study are listed in Supplementary Table 2.

Iterative staining of PNS biopsies was performed as described by Radtke et al.^61,62^. Staining and imaging were performed as described above. After imaging, the coverslip was removed and the slides washed 3× in PBS to remove any mounting medium. Bleaching of the fluorochromes was achieved using 1 mg ml^−1^ of a solution of lithium borohydride (Acros Organics, cat. no. 206810050) in water for 15 min at 20 °C. The slides were then washed 3× in PBS before staining as described above with a different set of antibodies. The process was repeated up to 7×. Raw imaging data were processed using Imaris with either Hoechst or CD19 as fiducial for alignment of subsequent images.

### Quantification and statistical analysis

#### ScRNA-seq data processing

Sequenced data were aligned to hu38 human reference genome using Cellranger v.6 (10x Genomics). Removal of ambient RNA and assigning of droplets were undertaken using Cellbender^63^. Automated doublet detection and filtering were performed using Scrublet^64^ (*P* < 0.1), as well as manually by gene co-expression. Quality control and filtering were performed in Scanpy^65^, using the sc-dandelion pre-processing module (dandelion.pp,recipe_scanpy_qc) with max. genes = 6000, min. genes = 200 and a Gaussian mixture model distribution to determine mitochondrial filtering values. Batch correction was performed with Harmony^66^ and Leiden clustering was also performed. Cell labels were assigned manually by gene expression profiles (Supplementary Fig.1c), with iterative rounds of subclustering to assign cell subtype labels. Assigned cluster labels were checked against signatures of flow-sorted, sequenced immune cells^67^ using SingleR^68^. Differential gene expression was performed by cell type and condition using FindMarkers in Seurat^69^ (logfc.threshold = 0.1, Min.pct = 0.1, min.cells.group = 10). GSEA^70^ was performed on lists of DEGs using fgsea^71^ (minSize = 15) and plotted in ggplot2, with *P* values calculated by permutation using the fgsea package intrinsic functions. Gene-set expression scores were calculated as the difference between the average expression levels of each gene set and randomly sampled pool of all (control) genes for each cell, using scanpy.tl.score_genes: an implementation of Seurat’s AddModuleScore function^72^. INFα response gene expression correlations were calculated using Pearson’s correlation coefficient implemented through the scipy package, with two-sided *P* values reported. Correlations were calculated on named cell subtypes with named gene expression values >0. Cell proportion plots were performed using ggplot2, with calculation of *P* values and confidence intervals (CIs) using a permutation and bootstrapping approach from the scProportionTest package. Trajectory analysis was performed using Slingshot^50^. For B cell trajectories, memory B cells, GC B cells and PCs were subset from the main object and reclustered. B cell Slingshot lineages were inferred with B GC light zone (B_GC_LZ) assigned as the starting cluster. For MNP cell trajectories, monocytes, macrophages and cDCs were subset from the main object and reclustered. MNP cell Slingshot lineages were inferred with classic monocytes assigned as the starting cluster. CellPhoneDB analysis was performed as previously described^73^, with additional plotting of results using ktplots. Single-cell TCR analysis was performed using Scirpy^74^. Single-cell BCR analysis was performed using sc-Dandelion^75^ with V(D)J gene reannotation and parsing to AIRR format performed in the sc-Dandelion singularity container (sc-dendelion_latest.sif). Gini’s index was calculated in sc-Dandelion, with *P* values calculated using Tukey’s honestly significant difference test implemented through the scikit-posthocs package. For identification of putative BCR sequences with capacity to bind SARS-CoV-2 surface antigens, we downloaded CDR3 sequences and germline assignments of known SARS-CoV-2-binding antibodies from the Coronavirus Antibody Database (CoV-AbDab, updated 26 July 2022)^48^ and filtered for antibodies of B cell origin. We defined putative SARS-CoV-2-binding BCR sequences in our dataset as B cells with complete CDR3-L match and significant motif similarity for CDR3-H compared with the CoV-AbDab database. Motif enrichment analysis between BCR CDR3-H sequences in our data and CoV-AbDab was performed using GLIPH2 (ref. ^36^). First, we compiled a reference database of naive BCR CDR3 sequences using published CDR3 sequence from COVID-naive B cells from multiple sources, for the GLIPH2 pipeline. Briefly, 1,437,743 CDR3-H sequences from bulk BCR sequencing (downloaded from iReceptor gateway 30 July 2022)^60^ and 55,444 CDR3-H sequences from three single-cell GEX + V(D)J-seq studies^75–77^ of control COVID-naive B cells were included in the reference. For GLIPH2 analysis, we considered only motifs that were more than six residues in length. We assessed motifs biased toward the target data (CoV-AbDab + BCR-seq V(D)J data combined) versus the naive reference data (Fisher’s exact test *P* < 0.05), to identify sequences enriched in our cohort. Shared motifs were visualized using ggseqlogo^78^. We recombined CDR3-H and CDR3-L sequences using their single-cell barcodes and filtered for single cells where both CDR3-H and CDR3-L sequences matched the corresponding CoV-AbDab antibody.

Harmonization and label transfer with a publicly available, airway brushing dataset were performed with scANVI^79^, using seed labels from the annotated NALT and PBMC sample cell-type labels^80–84^.

### Reporting summary

Further information on research design is available in the Nature Portfolio Reporting Summary linked to this article.

## Online content

Any methods, additional references, Nature Portfolio reporting summaries, source data, extended data, supplementary information, acknowledgements, peer review information; details of author contributions and competing interests; and statements of data and code availability are available at 10.1038/s41590-024-02064-9.

## Supplementary information

Supplementary Information Supplementary Fig. 1 and Tables 1 and 2.

Reporting Summary

## Source data

Source Data Statistical source data.

## Data Availability

All single-cell gene expression and V(D)J sequencing data have been uploaded to the Gene Expression Omnibus, accession no. (GSE287808) and will be freely available on publication. Source data are provided with this paper.

## Code availability

Details of packages and versions can be found in the online methods key resources table. Code to reproduce the analysis in the manuscript has been deposited in the Clatworthy lab GitHub repository (https://github.com/clatworthylab/COVID_analysis/COVID_NALT) and will be freely available on publication.
