# Supplementary material for: Temporal profiling of human lymphoid tissues reveals coordinated defense against viral challenge
Source: Nat Immunol. 2025 Jan 31;26(2):215–29. doi: 10.1038/s41590-024-02064-9 (PMC11785532; doi:10.1038/s41590-024-02064-9)
Supplement: Supplementary file 1 — Supplementary Fig. 1 and Tables 1 and 2. [file 41590_2024_2064_MOESM1_ESM.pdf]

nature immunology

Supplementary information

https://doi.org/10.1038/s41590-024-02064-9

Temporal profiling of human lymphoid tissues reveals coordinated defense against viral challenge

In the format provided by the authors and unedited

Supplementary information

Table of contents:

- Supplementary figure 1: Flow cytometry gating strategies.
- Supplementary table 1: Patient information
- Supplementary table 2: List of reagents used in this study

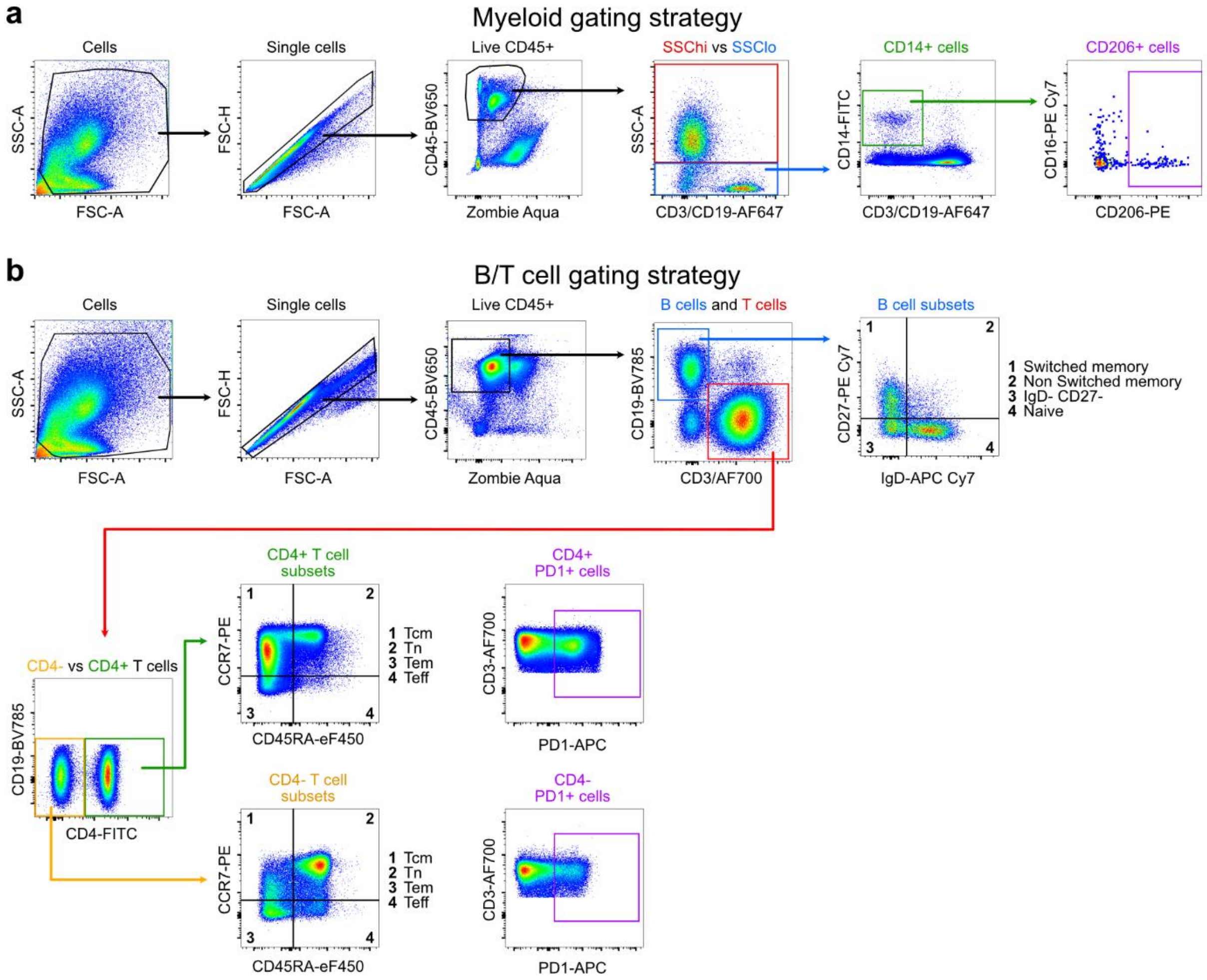

Supplementary Fig. 1

a, Gating strategy used to study myeloid cells by flow cytometry in Extended Data Fig. 2b and Extended Data Fig 4d. **b**, Gating strategy to study B and T cells by flow cytometry in Extended Data Fig 5c-e and Extended Data Fig. 7d-e. Tcm, central memory T cell; Tn, naïve T cell; Tem, effector memory T cell; Teff, effector T cell. Antibodies used can be found in Supplementary table 2.

Subject	Cohort	Gender	Age	Ethnicity	COVID severity	First positive PCR	Onset of symptoms	Admission to hospital for COVID	Co-morbidities	Smoking status	COVID therapy	Peak O2 req.	COVID diagnosis	COVID vaccine	PNS	Brush/MC	PBMC	Notes
1	Active COVID	Male	69	White-British	Severe	N/A	-11	-2	HTN	Ex-smoker	-	32%	CT	No	√	√	√	
2	Active COVID	Male	83	White-British	Moderate	-3	-7	-3	COPD, IHD, HTN, Asbestosis	Ex-smoker	CS, Baricitinib	35%	PCR	No	√	√	√	
3	Active COVID	Female	29	White-British	Mild/Asymp.	-1	N/A	N/A	None	Non-smoker	-	N/A	PCR	No	√		√	
4	Active COVID	Female	90	White-British	Severe	-2	-14	-2	HTN	Ex-smoker	CS, Remdesivir	60%	PCR	No	√	√	√	
5	Active COVID	Male	76	White-British	Severe	-1	-7	-1	HTN, DM, PVD	Ex-smoker	-	40%	PCR	No	√	√	√	
6	Active COVID	Male	22	White-British	Mild	-5	-6	N/A	None	Non-smoker	-	N/A	PCR	No	√		√	
7	Active COVID	Female	43	Asian-Other	Moderate	-6	-8	0	None	Non-smoker	CS	N/A	PCR	No	√		√	
8	Active COVID	Male	24	Asian-Chinese	Severe	-12	-13	-5	DM	Non-smoker	CS, Tocilizumab	40%	PCR	No	√			
9	Conval. COVID	Female	19	White-Other	Mild	-22	-19	N/A	None	Non-smoker	None	N/A	PCR	No	√	√	√	
10	Conval. COVID	Female	23	White-British	Mild	-21	-20	N/A	None	Non-smoker	None	N/A	PCR	No	√		√	
11	Conval. COVID	Female	24	White-British	Mild	-28	-28	N/A	None	Non-smoker	None	N/A	PCR	No	√		√	
12	Conval. COVID	Male	20	White-British	Mild/Asymp.	-26	N/A	N/A	None	Non-smoker	None	N/A	PCR	No			√	
13	Conval. COVID	Male	19	White-British	Mild	-28	-28	N/A	None	Non-smoker	None	N/A	PCR	No	√		√	
14	Healthy control	Male	23	White-British	N/A	N/A	N/A	N/A	None	Non-smoker	N/A	N/A	N/A	No	√		√	Sample pre-Dec 2019
15	Healthy control	Male	26	White-British	N/A	N/A	N/A	N/A	None	Non-smoker	N/A	N/A	N/A	No	√		√	Sample pre-Dec 2019
16	Healthy control	Male	35	White-British	N/A	N/A	N/A	N/A	None	Non-smoker	N/A	N/A	N/A	No	√			Sample pre-Dec 2019
17	Healthy control	Female	76	White-British	N/A	N/A	N/A	N/A	None	Non-smoker	N/A	N/A	N/A	No	√		√	Sample pre-Dec 2019
18	Healthy control	Male	91	White-British	N/A	N/A	N/A	N/A	PVD, AF	Smoker	N/A	N/A	N/A	No	√		√	Sample pre-Dec 2019
19	Healthy control	Male	85	White-British	N/A	N/A	N/A	N/A	None	Ex-smoker	N/A	N/A	N/A	No	√		√	Sample pre-Dec 2019
20	Healthy control	Male	30	Asian-Other	N/A	N/A	N/A	N/A	None	Non-smoker	N/A	N/A	N/A	Yes		√		No previous symptomatic COVID-19 infection
21	Healthy control	Female	23	White-Other	N/A	N/A	N/A	N/A	None	Non-smoker	N/A	N/A	N/A	Yes		√		Lateral flow negative
22	Healthy control	Female	32	White-Other	N/A	N/A	N/A	N/A	None	Non-smoker	N/A	N/A	N/A	Yes		√		Lateral flow negative
23	Healthy control	Female	58	Black-African	N/A	N/A	N/A	N/A	None	Non-smoker	N/A	N/A	N/A	Yes		√		Lateral flow negative

Supplementary table 1

Table showing characteristics of included subjects. COVID severity scoring by WHO criteria 1. AF, Atrial Fibrillation; Brush, COPD, Chronic Obstructive Pulmonary Disease; CS, Corticosteroids; CT, Computed Tomography scan; DM, Diabetes Mellitus; HTN, Hypertension; Brush, Nasal brushing of inferior turbinate; PBMC, Peripheral blood mononuclear cells; MC, Micro-curettage (of inferior nasal turbinate); PCR, SARS-CoV-2 RT-PCR nasopharyngeal swab test; PNS, Postnasal space; PVD, Peripheral Vascular Disease; N/A, Not applicable.

KEY RESOURCES TABLE		
REAGENT or RESOURCE	SOURCE	IDENTIFIER
Antibodies – Flow cytometry		
CD4 FITC RRID:AB_1659694	eBioscience	11-0049-42
CD69 PerCP-Cy5.5 RRID:AB_2074956	Biolegend	310926
PD1 APC RRID:AB_940473	Biolegend	329907
CD3 Alexa-Fluor700 RRID:AB_906220	eBioscience	56-0038-82
IgD APC/Cy7 RRID:AB_11204072	Biolegend	348217
CD45RA eFluor-450 RRID:AB_1272129	eBioscience	48-0458-41
Live/Dead Aqua	eBioscience	L34957
CD38 BV605 RRID:AB_2561527	Biolegend	303531
CD45 BV650 RRID:AB_2563812	Biolegend	304044
CD19 BV785 RRID:AB_11218596	Biolegend	302239
CCR7 PE RRID:AB_10916391	Biolegend	353203
CD27 PE-Cy7 RRID:AB_1724039	eBioscience	25-0279-42
CD14 FITC RRID:AB_10597597	eBioscience	11-0149-42
CD24 PerCP-Cy5.5 RRID:AB_10960741	Biolegend	311116
CD3 Alexa-Fluor-647 RRID:AB_389332	Biolegend	300416
CD19 Alexa-Fluor-647 RRID:AB_492935	Biolegend	302222
CD11b Alexa-Fluor-700 RRID:AB_2750074	Biolegend	301355
CD11c BV605 RRID:AB_2562191	Biolegend	301635
CD206 PE RRID:AB_10804655	Invitrogen	12-2069-42
CD16 PE-Cy7 RRID:AB_10714839	eBioscience	25-0168-42
Antibodies – Confocal microscopy		
CD3 eF450 1/50 RRID:AB_1518798	Invitrogen	48-0038-42
CD3 AF488 1/100 RRID:AB_389310	BioLegend	300415
CD4 AF647 1/100 RRID:AB_493097	BioLegend	300523
CD8 PE 1/100 RRID:AB_1953243	BioLegend	344705
CD11c PE 1/50 RRID:AB_10597432	Invitrogen	12-0116-42
CD11c APC 1/100 RRID:AB_2726453	Miltenyi	130-114-102
CD14 PE 1/25 RRID:AB_10598367	Invitrogen	12-0149-42
CD19 AF594 1/50 RRID:AB_2563233	BioLegend	302250
CD21 FITC 1/100 RRID:AB_2561574	BioLegend	354909
CD103 1/50 RRID:AB_11142856	Abcam	ab129202
CD138 AF647 1/100 RRID:AB_2564251	BioLegend	356524
CD163 AF647 1/100 RRID:AB_2563474	BioLegend	333619
CD206 AF488 1/100 RRID:AB_2574416	Invitrogen	53-2069-42
CitH3 1/100 RRID:AB_304752	Abcam	ab5103
EpCAM Pacific Blue 1/50 RRID:AB_10642820	BioLegend	324217
EpCAM eF660 1/100 RRID:AB_10598658	Invitrogen	50-9326-42
FolRb PE 1/50 RRID:AB_2721335	BioLegend	391703
Hoechst 33258 1/5000	Biotium	40044
IgA2 AF488 1/400 RRID:AB_2796665	Southern Biotech	9140-30
IgD AF488 1/25 RRID:AB_11150595	BioLegend	348216
IgG PE 1/50 RRID:AB_10900424	BioLegend	409303
Myeloperoxidase 1/100 RRID:AB_448948	Abcam	ab25989
PD-1 APC 1/50 RRID:AB_940473	BioLegend	329907

pIgR 1/50 RRID:AB_2878974	Proteintech	22024-1-AP
S100A9 FITC 1/50 RRID:AB_10662414	BioLegend	350703
Spike glycoprotein 1/500 RRID:AB_2847845	Abcam	ab272504
Donkey anti-mouse AF555 1/250 RRID:AB_2762848	Invitrogen	A32773
Donkey anti-rabbit AF555 1/300 RRID:AB_2762834	Invitrogen	A32794
Donkey anti-rabbit AF647 1/200 RRID:AB_2762835	Invitrogen	A32795
Biological samples		
Human Nasal Associated Lymphoid Tissue samples	This paper	
Human PBMCs	This paper	
Human Inferior Turbinate nasal brushing samples	This paper	
Chemicals, peptides, and recombinant proteins		
Brilliant stain buffer	BD Biosciences	566349
RPMI 1640 Medium with L-glutamine	Sigma	R8758
Histopaque 1077	Sigma	10771
LIberase TM	Sigma	05401127001
DNAse I	Roche	10104159001
Percoll	Sigma	P4937
AntigenFix	Diapath	P0016
OCT	Cellpath	KMA-0100-00A
FcR-Blocking Reagent	Miltenyi	130-059-901
Triton X-100	Sigma	93426-100ML
Fluoromount-G	Southern Biotech	0100-01
Normal mouse serum	Thermofisher	10410
Normal donkey serum	Abcam	ab7475
Lithium Borohydride	Acros Organics	206810050
Critical commercial assays		
Chromium Next GEM Single Cell V(D)J Reagent Kit v1.1	10x Genomics	1000165<br>1000005<br>1000016<br>1000120<br>1000213
Deposited data		
GEO Accession number	Upload in progress	
Software and algorithms		
CellphoneDB v2.1.7	[61]	https://github.com/Teichlab/cellphonedb
Cellranger v6.0.1	10x Genomics	https://support.10xgenomics.com/single-cell-gene-expression/software/downloads/
Fgsea 1.16.0	[62]	http://bioconductor.org/packages/release/bioc/html/fgsea.html
FlowJo v10	BD	https://www.flowjo.com/solutions/flowjo

Ggseqlogo v0.1	[63]	https://github.com/omarwagih/ggseqlogo
GLIPH2	[36]	http://50.255.35.37:8080/
Harmonypy v0.0.5	[64]	https://github.com/slowkow/harmonypy
Imaris v9	Oxford Instruments	https://imaris.oxinst.com/
Ktplots v1.1.14		https://github.com/zktuong/ktplots
Leidenalg v0.8.7	[65]	https://github.com/vtraag/leidenalg
Matplotlib v3.4.3		https://matplotlib.org/
Msigdbr 7.4.1		https://github.com/igordot/msigdbr
Pandas 1.3.2		https://pandas.pydata.org/
Prism v9	Graphpad software	https://www.graphpad.com/scientific-software/prism/
Python v3.8		https://www.python.org/
R v4.0.4		https://www.r-project.org/
Reticulate v1.20		https://github.com/rstudio/reticulate
Sc-dandelion v0.1.11	[66]	https://sc-dandelion.readthedocs.io/en/latest/
Scanpy v1.8.1	[67]	https://github.com/scverse/scanpy
scANVI v0.19.0	[68]	https://docs.scvi-tools.org/en/stable/
Sceasy v0.0.6		https://github.com/cellgeni/sceasy
Scikit-posthocs v0.6.7		https://scikit-posthocs.readthedocs.io/en/latest/
Scipy v1.7.1		https://scipy.org/
Scirpy v0.10.1	[69]	https://github.com/scverse/scirpy
scProportionTest v0.0.0.9000		https://github.com/rpolicastro/scProportionTest
Scrublet v0.2.3	[70]	https://github.com/swolock/scrublet
Seaborn v0.11.2	[71]	https://seaborn.pydata.org/

Seurat v4.0.1	[72]	https://satijalab.org/seurat/
SingleCellExperiment v1.12.0	[73]	https://bioconductor.org/packages/release/bioc/html/SingleCellExperiment.html
SingleR	[74]	https://github.com/dviraran/SingleR
Slingshot v1.8.0	[50]	https://www.bioconductor.org/packages/release/bioc/html/slingshot.html
tidyverse v1.3.1		https://www.tidyverse.org/
Other		
Code repository for single cell RNA sequencing analysis	This paper	https://github.com/clatworthylab/COVID_analysis/COVID_NALT

Supplementary table 2

Reagents and resources used throughout this publication.
